# Associations of Novel Dietary and Lifestyle Inflammation Scores With Incident Colorectal Cancer in the NIH-AARP Diet and Health Study

**DOI:** 10.1093/jncics/pkaa009

**Published:** 2020-03-05

**Authors:** Doratha A Byrd, Suzanne E Judd, W Dana Flanders, Terryl J Hartman, Veronika Fedirko, Tanya Agurs-Collins, Roberd M Bostick

**Affiliations:** p1 Department of Epidemiology, Emory University Rollins School of Public Health, Atlanta, GA, USA; p2 Department of Biostatistics, University of Alabama, Birmingham School of Public Health, Birmingham, AL, USA; p3 National Cancer Institute, National Institutes of Health, Bethesda, MD, USA; p4 Winship Cancer Institute, Emory University, Atlanta, GA, USA

## Abstract

**Background:**

Chronically higher inflammation, likely contributed to by dietary and lifestyle exposures, may increase risk for colorectal cancer (CRC). To address this, we investigated associations of novel dietary (DIS) and lifestyle (LIS) inflammation scores with incident CRC in the prospective National Institutes of Health–American Association of Retired Persons Diet and Health Study (N = 453 465).

**Methods:**

The components of our previously developed and externally validated 19-component DIS and 4-component LIS were weighted based on their strengths of associations with a panel of circulating inflammation biomarker concentrations in a diverse subset (N = 639) of participants in the REasons for Geographic and Racial Differences in Stroke Study cohort. We calculated the components and applied their weights in the National Institutes of Health-American Association of Retired Persons cohort at baseline, summed the weighted components (higher scores reflect a higher balance of proinflammatory exposures), and investigated associations of the scores with incident CRC using Cox proportional hazards regression. All statistical tests were two-sided.

**Results:**

Over a mean 13.5 years of follow-up, 10 336 participants were diagnosed with CRC. Among those in the highest relative to the lowest DIS and LIS quintiles, the multivariable-adjusted hazards ratios (HRs) and their 95% confidence intervals (CIs) were HR = 1.27 (95% CI = 1.19 to 1.35; *P*_trend_ < .001) and 1.38 (95% CI = 1.30 to 1.48; *P*_trend_ < .001), respectively. The associations were stronger among men and for colon cancers. The hazards ratio for those in the highest relative to the lowest joint DIS and LIS quintile was HR = 1.83 (95% CI = 1.68 to 1.99; *P*_interaction_ < .001).

**Conclusions:**

Aggregates of proinflammatory dietary and lifestyle exposures may be associated with higher risk for CRC.

Although inflammation is normal, chronically higher amounts may be harmful and contribute to the development of chronic diseases and cancer, especially colorectal cancer (CRC) (1–4), the second leading cause of cancer death in the United States (5). Inflammation promotes colorectal carcinogenesis by damaging DNA and increasing cell proliferation and angiogenesis (6–12). CRC is also highly associated with diet and lifestyle factors that may be chronic inflammation sources (13–15).

The contributions of individual dietary components to systemic inflammation are likely small but collectively may be substantial. To address this, several dietary inflammation scores were developed, such as the dietary inflammatory index (DII), to characterize the aggregate contributions of dietary exposures to systemic inflammation. In the National Institutes of Health–American Association of Retired Persons Diet and Health Study (NIH-AARP), a large prospective cohort study of older US adults, the DII was modestly, statistically significantly associated with higher CRC risk among men but not women (16). Importantly, the DII has some limitations, including that its heavy focus on selected nutrients may not account for many other known and unknown dietary constituents that may affect inflammation. There are no published lifestyle-specific inflammation scores.

Accordingly, we previously developed novel, inflammation biomarker panel-weighted lifestyle inflammation scores (LIS) and predominantly whole-foods based dietary inflammation scores (DIS) and validated their constructions in three populations (17). Herein, we report an investigation of associations of the DIS and LIS with incident CRC in the NIH-AARP study.

## Methods

### Study Population

NIH-AARP (18) is a large prospective cohort study designed to investigate diet–cancer associations. It was approved by the Special Studies Institutional Review Board of the US National Cancer Institute. From 1995 to 1996, a self-administered questionnaire was mailed to 3.5 million 50- to 71-year-old adults in 6 US states (California, Florida, Louisiana, New Jersey, North Carolina, and Pennsylvania) and two metropolitan areas (Atlanta, GA and Detroit, MI) (17.6% response rate).

A supplementary Risk Factor Questionnaire, which included questions on aspirin and other nonsteroidal anti-inflammatory drug (NSAID) use, was mailed 6 months after baseline. A follow-up questionnaire, which included questions on cancer screening, was mailed in 2004–2005.

A total of 566 398 respondents completed the baseline questionnaire. Briefly, we excluded from analysis those with a history of cancer or end-stage renal disease; death-only classification of CRC or other cancers; missing responses for over 15% of questions on the Diet History Questionnaire (DHQ); implausible energy intakes (<500 or >6000 kcal/d); or missing lifestyle questions (see details in Supplementary Methods, available online). The final analytic sample size was 453 465.

### Data Collection

Mailed questionnaires included a detailed 124-item, grid-based version of the NCI DHQ that measured usual diet over the past year and was validated against two 24-hour dietary recalls (via telephone, 25 days apart) in a subset of 2000 cohort participants (19–21). Ten possible frequency-of-consumption responses, ranging from “never” to “6+ times per day” were given for each food item. The DHQ also ascertained frequencies of alcohol and supplemental micronutrient intakes. Energy and nutrient intakes were calculated using the nutrient composition database derived from the US Department of Agriculture Continuing Survey of Food Intakes by Individuals national survey data. Selected food group intakes (eg, dairy and tomato foods) were calculated using the MyPyramid Equivalents Database, as described previously (22,23). The questionnaire also ascertained self-reported smoking status, weight, height, and frequency of physical activity lasting at least 20 minutes that is intense enough to work up a sweat or increase breathing or heart rate.

### Outcome Ascertainment

Incident CRC cases were identified using probabilistic linkage of the cohort participants to cancer registries of the states where participants resided at baseline, and 3 states (Arizona, Texas, and Nevada) to which participants were most likely to move during follow-up. The registry validly identified approximately 90% of cancer cases (24). We defined incident CRC cases according to *International Classification of Diseases for Oncology* codes (Supplementary Methods, available online), right colon as extending from the cecum through the transverse colon, and left colon as the splenic flexure through the sigmoid colon.

### DIS and LIS Descriptions

The development and validation of the DIS and LIS were described previously (17) (also Supplementary Methods, available online). Briefly, the 19 and 4 components of the DIS and LIS, respectively, were determined and grouped a priori, based on previous literature and their expected contributions to systemic inflammation and ease of reconstruction in major epidemiologic studies, using Block 98 food frequency questionnaire (25) and lifestyle questionnaire responses (Supplementary Table 1, available online) in a diverse subset (N = 639) of participants in the REasons for Geographic and Racial Differences in Stroke Study cohort (REGARDS) (26,27). Weights for the DIS and LIS components (Table 1) were calculated in REGARDS based on their multivariable-adjusted strengths of associations with an inflammation biomarker score comprising high-sensitivity C-reactive protein, interleukin (IL)-6, IL-8, and IL-10.

**Table 1.** Components and weights of the DIS and LIS and their descriptions in the NIH-AARP

Components	Descriptions	Weights*
DIS components^†^		
Leafy greens and cruciferous vegetables	Cooked or raw spinach, kale, lettuce salad, broccoli, cabbage or coleslaw, cauliflower, Brussels sprouts, and turnip, collard, or mustard greens	–0.14
Tomatoes	Tomatoes, tomato juice, tomato sauce, salsa, and tomato or spaghetti sauce	–0.78
Apples and berries	Apples, applesauce, pears, and strawberries	–0.65
Deep yellow or orange vegetables and fruit	Peaches, nectarines, plums, cantaloupe, and carrots	–0.57
Other fruits and real fruit juices	Watermelon, oranges, tangerines, tangelos, grapefruit, other melon (eg, watermelon or honeydew), grapes, orange juice, grapefruit juice, and other fruit juice	–0.16
Other vegetables	Sweet peppers (green or red)	–0.16
Legumes	String beans, green beans, peas, and beans	–0.04
Fish	Tuna and other fried or nonfried fish	–0.08
Poultry	Ground chicken or turkey, roast turkey, turkey cutlets, turkey nuggets, fried chicken or chicken nuggets, and baked, broiled, roasted, or stewed chicken	–0.45
Red and organ meats	Ground beef, roast beef, steak, roast ham, ham steak, pork chops, pork roasts, and liver or liverwursts	0.02
Processed meats	Hot dogs, frankfurters, bacon, sausage, bologna, salami, corned beef, pastrami, turkey or chicken cold cuts/luncheon meats	0.68
Added sugars	Hi-C, Kool-Aid, lemonade, soda, dried fruit, chocolate candy, and other candy	0.56
High-fat dairy	Whole milk, full-fat cottage cheese, full-fat yogurt, cream cheese, sour cream, full-fat cheese or cheese spreads, and full-fat ice cream or ice bars	–0.14
Low-fat dairy	Low-fat frozen yogurt, skim milk, low-fat cottage cheese, low- or reduced-fat cheese; low-fat ice cream, ice milk, or sherbet; and skim, 1%, or 2% milk	–0.12
Coffee and tea	Iced or hot tea and regular or decaf coffee	–0.25
Nuts	Peanut butter, other nut butter, peanuts, walnuts, seeds, and other nuts	–0.44
Other fats	Butter, margarine, mayonnaise, meat gravy, lard, vegetable shortening, and liquid oil (corn, canola)	0.31
Refined grains and starchy vegetables	Cake, cookies, brownies, doughnuts, sweet rolls, Danish, sweet muffins, dessert breads, fruit pie, cream custard or meringue pie, pumpkin or sweet potato pie, pancakes, waffles, French toast, crepes, bran cereal, fiber and nonfiber cereals, French fries, home fries, hash brown potatoes, potato salad, rice, pasta, spaghetti, other noodles, bagels, English muffins, breads, rolls, crackers, cornbread, muffins, biscuits, flour or corn tortillas, potato chips, sweet potatoes or yams, baked, boiled, or mashed potatoes; oatmeal, grits, or other cooked cereals	0.72
Supplement score^‡^	Ranked score of supplements, including multivitamins, zinc, iron, selenium, folic acid, calcium, β-carotene, and vitamins A, C, and E	–0.80
LIS components^†^		
Heavy drinker	Heavy (>7 drinks/wk for women, >14 drinks/wk drinks for men) vs nondrinker	0.30
Moderate drinker	Moderate (1–7 drinks/wk for women, 1–14 drinks/wk for men) vs nondrinker	–0.66
Moderately physically active	Exercises 1–3 times/mo or 1–2 times/wk vs never or rarely exercises	–0.18
Heavily physically active	Exercises ≥3 times/wk vs never or rarely exercises	–0.41
Current smoker	Currently smoked tobacco at baseline vs did not currently smoke tobacco	0.50
Overweight BMI	Overweight BMI (25–29.99 kg/m^2^) vs normal BMI (18.5–24.99 kg/m^2^)	0.89
Obese BMI	Obese BMI (≥30 kg/m^2^) vs normal BMI (18.5–24.99 kg/m^2^)	1.57

* Weights are β coefficients from multivariable linear regression models conducted in a subset of the REGARDS cohort (N = 639) and represent the average change in an inflammation biomarker score (sum of z scores for high-sensitivity C-reactive protein, IL-6, IL-8, and IL-10 [the latter with a negative sign]) per 1-SD increase in a dietary component or the presence of a lifestyle component. Covariates in the final model to develop the weights included: age, sex, race (black or white), education (high school graduate or less vs some college or more), region (stroke belt, stroke buckle, or other region in the United States), a comorbidity score (comprises a history of cancer, heart disease, diabetes mellitus, or chronic kidney disease), HRT (among women), total energy intake (kcal/d), season of baseline interview (spring, summer, fall, or winter), and regular use of aspirin, other NSAIDs, or lipid-lowering medications (≥ twice/wk); and all the dietary and lifestyle components in the DIS and LIS. For the NIH-AARP study, all dietary components were standardized based on the sex-specific distribution in the analytic cohort at baseline, and all lifestyle components were dummy variables. BMI = body mass index; DIS = dietary inflammation score; HRT = hormone replacement therapy; hsCRP = high sensitivity C-reactive protein; IL = interleukin; LIS = lifestyle inflammation score; NIH-AARP = National Institutes of Health–American Association for Retired Person Diet and Health Study; REGARDS = REasons for Geographic and Racial Differences in Stroke study.

† Components listed are based on food and lifestyle items measured in the baseline NIH-AARP Dietary Health Questionnaire.

‡ All vitamin and mineral supplement intakes measured (from multivitamin and mineral and individual supplements) were ranked into quantiles of intake and assigned a value of 0 (low or no intake), 1, or 2 (highest intake) for hypothesized anti-inflammatory supplements (eg, selenium), and 0 (low or no intake), –1, or –2 (highest intake) for hypothesized proinflammatory supplements (eg, iron).

### DIS and LIS Calculations in NIH-AARP

The DIS and LIS components were constructed in the NIH-AARP cohort based on DHQ responses as summarized in Table 1. We used MyPyramid Equivalents Database food group equivalents to disaggregate mixed dishes into their component parts (eg, tomatoes in pizza) and added the equivalents to their appropriate DIS groups. To account for supplemental micronutrients, we calculated a supplement score (described in Table 1). We standardized each DIS component, by sex, based on the study baseline distribution. Because all LIS components were categorical (see Table 1; Supplementary Methods, available online), we created indicator variables (1 or 0) for each lifestyle characteristic compared with the referent group.

Next, the value for each NIH-AARP participant’s DIS and LIS component was multiplied by its respective weight that was calculated in the REGARDS development population. Finally, the weighted values for each participant’s score components were summed to constitute their DIS or LIS. Higher scores indicate a higher balance of pro- to anti-inflammatory exposures.

### Statistical Analyses

All analyses were conducted using SAS statistical software, version 9.3. All statistical tests were two-sided, and *P* less than .05 or 95% confidence intervals (CIs) that excluded 1.0 were considered statistically significant. Total follow-up time was calculated as years between completing the baseline questionnaire and the date of a participant’s first CRC diagnosis, date of death, date they moved from the catchment area, or the last study follow-up (December 31, 2011), whichever came first. Those noncontemporaneously diagnosed with both colon and rectal cancers were censored based on the date of whichever diagnosis came first.

We categorized participants into LIS and sex-specific DIS quintiles at baseline. We used Cox proportional hazards regression to estimate multivariable-adjusted hazards ratios (HRs) and 95% confidence intervals for associations of the DIS and LIS with incident CRC (overall and by colorectal site). Before Cox proportional hazards regression modeling, we assessed the proportional hazards assumption (see Supplementary Methods, available online). We assessed trend by entering the median of each inflammation score quintile into the multivariable Cox proportional hazards regression models as a continuous variable.

To assess potential interaction between the DIS and LIS, we conducted a joint and combined (cross-classification) analysis using multivariable Cox proportional hazards regression models in which the reference group was participants in the first quintile of both scores. We entered a categorical DIS × LIS term into the model to calculate a Wald *P*_interaction_.

Potential confounders were based on biological plausibility, previous literature, and causal diagrams. Final model covariates are listed in table footnotes. We also conducted separate analyses within categories of participant characteristics that could plausibly modify the DIS– or LIS–CRC associations (eg, baseline age, race), and calculated Wald test *P*_interaction_ terms (see Supplementary Methods, available online).

### Sensitivity Analyses 

To assess the sensitivity of the associations to various considerations (additional details in Supplementary Methods, available online), we repeated the analyses with the following variations. First, we constructed equally weighted DIS and LIS versions by assigning positive or negative equal weights to dietary and lifestyle components we hypothesized a priori to be proinflammatory or anti-inflammatory, respectively. Second, we calculated a DIS without supplemental micronutrients. Third, we calculated a reverse-direction Healthy Eating Index-2015 (ie, so a higher score would be higher risk) (28), and the empirical dietary inflammatory pattern (EDIP), as described by Tabung et al. (29), and investigated their associations with CRC. Fourth, we investigated associations of each individual lifestyle component with CRC. Fifth, we excluded individuals who died or were diagnosed with CRC within 2 years from baseline.

## Results

Over an average of 13.5 years of follow-up, 10 336 participants developed CRC (76.0% developed colon cancer, 22.1% rectal cancer, and 1.9% both colon and rectal cancer).

Selected baseline characteristics of the NIH-AARP analytic cohort according to DIS and LIS quintiles are presented in Table 2. Those in the highest (most proinflammatory) relative to the lowest DIS and LIS quintiles were more likely to be less formally educated, not use hormone replacement therapy (among women), be a current smoker, be overweight or obese, be a nondrinker, exercise less than 3 times/wk, and for the LIS, more likely to have a comorbidity. On average, those in the highest DIS and LIS quintiles had lower dietary fiber intakes and higher reverse-direction Healthy Eating Index-2015 and EDIP scores; for the DIS, lower total calcium intakes; and for the LIS, higher total energy intakes.

**Table 2.** Selected baseline characteristics of the NIH-AARP participants (N = 453 465) across quintiles of the DIS and LIS, 1995–2011*

	DIS quintile			LIS quintile		
Characteristics^†^	1 (N = 90 743)	3 (N = 90 744)	5 (N = 90 743)	1 (N = 91 994)	3 (N = 91 456)	5 (N = 82 198)
Score range	–14.9 to –2.0	–0.6 to 0.6	2.0 to 12.8	–1.1 to -0.7	–0.2 to 0.2	0.8 to 2.4
Demographics						
Age at entry, y	61.6 (5.3)	61.6 (5.4)	61 (5.5)	61.6 (5.4)	61.5 (5.4)	61.2 (5.3)
Male, %	59.9	59.9	59.9	54.5	59.7	53.6
White, %	93.1	92.6	89.5	93.2	92.4	90.1
College graduate higher, %	48.0	40.5	27.6	48.9	39.6	29.0
Marital status, %	67.8	70.1	68.8	68.7	69.9	63.6
Medical history						
No comorbidity^‡^, %	71.0	70.4	70.0	78.9	73.6	59.9
HRT user (women), %	49.6	46.8	36.8	55.2	47.6	34.7
Family history of CRC^‖^, %	9.1	8.9	8.3	9.2	8.9	8.6
Previously diagnosed with colon polyp, %	9.1	9.7	8.9	8.2	9.2	9.8
Lifestyle						
Current smoker, %	6.9	10.8	20.9	0.0	6.2	20.7
Normal BMI^¶^, %	38.6	34.9	33.5	100	33.9	1.4
Nondrinker, %	21.0	22.6	29.7	0.0	17.6	50
Exercises ≥3 times/wk, %	60.7	45.9	32.9	57.6	10.1	25.6
Dietary intakes						
Total energy, kcal/d	1917 (812)	1785 (767)	1924 (870)	1710 (674)	1789 (755)	2011 (960)
Carbohydrates, % kcal/d	55.7 (9.9)	52.1 (9.4)	50.3 (9.8)	55.1 (9.0)	51.8 (9.4)	49.4 (10.6)
Proteins, % kcal/d	16.3 (3.2)	15.5 (3.0)	14.3 (3.2)	15.3 (2.9)	15.2 (3.1)	15.1 (3.6)
Total fats, % kcal/d	26.8 (7.4)	30.5 (7.3)	33.1 (7.6)	28.6 (7.4)	30.4 (7.5)	31.1 (8.3)
Total calcium^#^, mg/d	896 (493)	757 (429)	705 (446)	753 (417)	739 (428)	793 (489)
Dietary fiber, g/1000 kcal/d	14.0 (4.2)	10.7 (3.2)	8.0 (2.7)	11.8 (4.0)	10.4 (3.6)	9.8 (3.7)
Reverse HEI-2015 score**	58.6 (9.1)	68.7 (7.7)	74.3 (6.9)	65.6 (9.7)	67.1 (9.5)	70 (9.1)
EDIP score^††^	–0.15 (0.4)	–0.05 (0.3)	0.05 (0.3)	–0.09 (0.3)	–0.06 (0.3)	–0.04 (0.4)

* Inflammation scores constructed as described in the text and Table 1; a higher score reflects a higher balance of proinflammatory exposures. BMI = body mass index; CRC = colorectal cancer; DIS = dietary inflammation score; EDIP = empirical dietary inflammatory pattern; HEI = Healthy Eating Index; HRT = hormone replacement therapy; LIS = lifestyle inflammation score; NIH-AARP = National Institutes of Health-American Association for Retired Persons Diet and Health Study; Reverse HEI-2015 = reverse-direction Healthy Eating Index-2015.

† Presented as means (SD) unless otherwise specified.

‡ Comprises self-reported baseline gallstone or gallbladder disease, emphysema, heart disease, or diabetes mellitus.

‖ In a first-degree relative.

¶ 18.5–24.99 kg/m^2^.

# Total = diet + supplements.

** Calculated as described in Krebs-Smith et al. (28) except the direction of the contributions of the components to the score were reversed, such that a higher reverse HEI-2015 score reflects lower rather than higher adherence to HEI recommendations (ie, so a higher score would be higher risk); the Spearman correlation coefficient between the Reverse HEI-2015 and the DIS is R_s_ = 0.59.

†† Calculated as described in Tabung et al. (29); the Spearman correlation coefficient between the EDIP and the DIS is R_s_ = 0.25.

Multivariable-adjusted associations of the DIS and LIS with incident CRC, overall, by tumor site, and by sex, are presented in Table 3. For those in the highest relative to the lowest DIS quintile, CRC risk was statistically significantly 27% (95% CI = 19% to 35%) higher. For men and women, the DIS was similarly directly associated with right and left colon cancers, and risks for colon and rectal cancers were statistically significantly 29% and 21% higher, respectively.

**Table 3.** Associations of the DIS and LIS* with incident CRC overall and by sex and CRC site; the NIH-AARP, 1995–2011 (N = 453 465)

	Overall				Men^†^				Women^†^			
Cancer site		DIS^‡^		LIS^‖^		DIS^‡^		LIS^‖^		DIS^‡^		LIS^‖^
	No. cases	Adjusted HR (95% CI)	No. cases	Adjusted HR (95% CI)	No. cases	Adjusted HR (95% CI)	No. cases	Adjusted HR (95% CI)	No. cases	Adjusted HR (95% CI)	No. cases	Adjusted HR (95% CI)
Colorectal												
Continuous		1.04 (1.03 to 1.05)		1.16 (1.13 to 1.19)		1.04 (1.03 to 1.05)		1.20 (1.15 to 1.24)		1.03 (1.02 to 1.05)		1.10 (1.05 to 1.15)
Quintiles												
1	1877	1.00 (Referent)	1727	1.00 (Referent)	1243	1.00 (Referent)	1052	1.00 (Referent)	634	1.00 (Referent)	675	1.00 (Referent)
2	1905	1.01 (0.95 to 1.08)	1978	1.13 (1.05 to 1.20)	1297	1.04 (0.96 to 1.12)	1454	1.15 (1.06 to 1.24)	608	0.96 (0.86 to 1.08)	524	1.11 (0.99 to 1.25)
3	2008	1.06 (0.99 to 1.13)	2155	1.21 (1.14 to 1.29)	1340	1.07 (0.99 to 1.15)	1477	1.29 (1.19 to 1.39)	668	1.04 (0.93 to 1.16)	678	1.08 (0.97 to 1.20)
4	2126	1.11 (1.05 to 1.19)	2315	1.22 (1.15 to 1.30)	1397	1.10 (1.02 to 1.19)	1588	1.26 (1.16 to 1.36)	729	1.13 (1.01 to 1.27)	727	1.16 (1.04 to 1.30)
5	2420	1.27 (1.19 to 1.35)	2161	1.38 (1.30 to 1.48)	1628	1.29 (1.19 to 1.39)	1334	1.49 (1.37 to 1.62)	792	1.21 (1.08 to 1.36)	827	1.22 (1.10 to 1.36)
*P*_trend_		<.001		<.001		<.001		<.001		<.001		<.001
Colon^¶^												
Continuous		1.04 (1.03 to 1.05)		1.19 (1.16 to 1.23)		1.05 (1.04 to 1.06)		1.24 (1.19 to 1.29)		1.03 (1.02 to 1.05)		1.13 (1.07 to 1.18)
Quintiles												
1	1466	1.00 (Referent)	1294	1.00 (Referent)	955	1.00 (Referent)	775	1.00 (Referent)	511	1.00 (Referent)	519	1.00 (Referent)
2	1464	1.00 (0.93 to 1.07)	1530	1.17 (1.08 to 1.26)	979	1.01 (0.93 to 1.11)	1120	1.20 (1.10 to 1.32)	485	0.97 (0.85 to 1.10)	410	1.13 (0.99 to 1.29)
3	1536	1.04 (0.96 to 1.12)	1669	1.26 (1.17 to 1.35)	1017	1.06 (0.96 to 1.15)	1117	1.33 (1.21 to 1.46)	519	1.01 (0.89 to 1.14)	552	1.14 (1.01 to 1.29)
4	1672	1.13 (1.05 to 1.21)	1844	1.31 (1.22 to 1.41)	1084	1.12 (1.02 to 1.22)	1255	1.36 (1.24 to 1.49)	588	1.15 (1.01 to 1.30)	589	1.22 (1.08 to 1.38)
5	1911	1.29 (1.20 to 1.38)	1712	1.47 (1.36 to 1.59)	1275	1.33 (1.22 to 1.45)	1043	1.60 (1.45 to 1.76)	636	1.21 (1.07 to 1.38)	669	1.29 (1.14 to 1.45)
*P*_trend_		<.001		<.001		<.001		<.001		.001		.01
Left colon^#^												
Continuous		1.04 (1.03 to 1.06)		1.23 (1.17 to 1.30)		1.05 (1.03 to 1.07)		1.29 (1.21 to 1.37)		1.02 (0.99 to 1.05)		1.14 (1.04 to 1.25)
Quintiles												
1	512	1.00 (Referent)	432	1.00 (Referent)	357	1.00 (Referent)	274	1.00 (Referent)	155	1.00 (Referent)	158	1.00 (Referent)
2	534	1.04 (0.92 to 1.17)	527	1.17 (1.03 to 1.33)	380	1.03 (0.89 to 1.20)	403	1.21 (1.04 to 1.41)	154	1.04 (0.83 to 1.31)	124	1.12 (0.89 to 1.42)
3	557	1.08 (0.95 to 1.22)	628	1.38 (1.22 to 1.56)	387	1.06 (0.91 to 1.22)	451	1.48 (1.27 to 1.72)	170	1.14 (0.91 to 1.43)	177	1.20 (0.97 to 1.49)
4	596	1.12 (0.99 to 1.27)	699	1.43 (1.26 to 1.61)	409	1.09 (0.95 to 1.27)	503	1.49 (1.28 to 1.73)	187	1.20 (0.96 to 1.51)	196	1.32 (1.07 to 1.64)
5	724	1.33 (1.18 to 1.50)	637	1.59 (1.40 to 1.80)	523	1.37 (1.19 to 1.58)	425	1.75 (1.50 to 2.05)	201	1.24 (0.99 to 1.56)	212	1.32 (1.06 to 1.64)
*P*_trend_		<.001		<.001		<.001		<.001		.03		.002
Right colon**												
Continuous		1.05 (1.03 to 1.06)		1.17 (1.12 to 1.22)		1.05 (1.03 to 1.07)		1.21 (1.15 to 1.28)		1.04 (1.02 to 1.06)		1.12 (1.05 to 1.19)
Quintiles												
1	858	1.00 (Referent)	792	1.00 (Referent)	528	1.00 (Referent)	455	1.00 (Referent)	330	1.00 (Referent)	337	1.00 (Referent)
2	842	0.99 (0.90 to 1.09)	902	1.15 (1.04 to 1.26)	539	1.03 (0.91 to 1.16)	643	1.18 (1.05 to 1.34)	303	0.93 (0.79 to 1.09)	259	1.1 (0.93 to 1.29)
3	880	1.02 (0.93 to 1.12)	937	1.17 (1.06 to 1.29)	564	1.08 (0.95 to 1.21)	594	1.22 (1.08 to 1.38)	316	0.93 (0.80 to 1.09)	343	1.09 (0.94 to 1.27)
4	985	1.15 (1.05 to 1.27)	1034	1.23 (1.12 to 1.35)	609	1.16 (1.03 to 1.31)	668	1.26 (1.12 to 1.43)	376	1.14 (0.98 to 1.33)	366	1.18 (1.01 to 1.37)
5	1083	1.30 (1.18 to 1.43)	983	1.40 (1.27 to 1.55)	680	1.35 (1.20 to 1.52)	560	1.52 (1.34 to 1.73)	403	1.22 (1.04 to 1.42)	423	1.26 (1.08 to 1.46)
*P*_trend_		<.001		<.001		<.001		<.001		.001		<.001
Rectum or rectosigmoid												
Continuous		1.03 (1.01 to 1.04)		1.05 (0.99 to 1.11)		1.03 (1.00 to 1.05)		1.06 (0.99 to 1.14)		1.02 (0.99 to 1.05)		1.01 (0.92 to 1.11)
Quintiles												
1	450	1.00 (Referent)	462	1.00 (Referent)	313	1.00 (Referent)	293	1.00 (Referent)	137	1.00 (Referent)	169	1.00 (Referent)
2	475	1.04 (0.91 to 1.19)	489	1.02 (0.90 to 1.16)	343	1.09 (0.94 to 1.28)	369	1.04 (0.89 to 1.21)	132	0.91 (0.71 to 1.16)	120	1.02 (0.81 to 1.29)
3	501	1.08 (0.95 to 1.23)	531	1.10 (0.97 to 1.24)	348	1.09 (0.93 to 1.27)	398	1.22 (1.05 to 1.43)	153	1.06 (0.84 to 1.34)	133	0.84 (0.67 to 1.06)
4	490	1.05 (0.92 to 1.20)	523	1.01 (0.89 to 1.15)	337	1.05 (0.90 to 1.23)	372	1.03 (0.88 to 1.21)	153	1.04 (0.82 to 1.32)	151	0.97 (0.77 to 1.21)
5	568	1.21 (1.06 to 1.38)	479	1.13 (0.99 to 1.29)	398	1.21 (1.04 to 1.42)	307	1.18 (1.00 to 1.40)	170	1.18 (0.93 to 1.50)	172	1.02 (0.81 to 1.28)
*P*_trend_		.01		.15		.04		.09		.10		.99

* Inflammation scores constructed as described in the text and Table 1; a higher score reflects a higher balance of proinflammatory exposures. BMI = body mass index; CI = confidence interval; CRC = colorectal cancer; DIS = dietary inflammation score; HR = hazards ratio; HRT = hormone replacement therapy; LIS = lifestyle inflammation score; NIH-AARP = National Institutes of Health-American Association for Retired Persons Diet and Health Study.

† *P*_interaction_ comparing DIS associations between men and among women was >.05 for each cancer subsite; *P*_interaction_ comparing LIS associations between men and women was .01, .03, and .04 for colorectal, colon, and rectal cancer sites, respectively, but >.05 for all other cancer sites.

‡ Covariates in the DIS Cox proportional hazards models were age at entry (continuous), sex, race (black, white, or other), education (less than high school and high school graduate, some college, or college graduate or higher), marital status (married or nonmarried), heart disease or history of stroke at baseline (yes or no), diabetes mellitus at baseline (yes or no), emphysema at baseline (yes or no), gallstone or gallbladder disease at baseline (yes or no), current HRT use (among women), family history of CRC in a first-degree relative, history of colon polyp, smoking (current, former, or never), BMI (in kg/m^2^; continuous), alcohol intake (nondrinker, moderate drinker, or heavy drinker), physical activity level (exercises not at all or rarely, 1–2 times/wk, or ≥3 times/wk), and total energy intake (kcal/d); history of CRC in a first-degree relative, self-reported heart disease diagnosis, age at entry, sex, and BMI were included in the SAS *STRATA* statement.

‖ Covariates in the LIS Cox proportional hazards models were age at entry (continuous), sex, race (black, white, or other), education (less than high school and high school graduate, some college, or college graduate or higher), marital status (married or nonmarried), heart disease or history of stroke at baseline (yes or no), diabetes mellitus at baseline (yes or no), emphysema at baseline (yes or no), gallstone or gallbladder disease at baseline (yes or no), current HRT use (among women), family history of CRC in a first-degree relative, history of colon polyp, total energy intake (kcal/d), former smoker (yes or no), and the equally weighted DIS; history of CRC in a first-degree relative, self-reported heart disease diagnosis, age at entry, and sex were included in the SAS *STRATA* statement.

¶ The *P*_heterogeneity_ for colon vs rectum or rectosigmoid associations was .01 and <.0001 for the overall DIS and LIS models, respectively.

# Splenic flexure, descending, sigmoid; the *P*_heterogeneity_ for left colon vs rectum or rectosigmoid associations was .39 and <.0001 for the overall DIS and LIS models, respectively.

** Cecum, hepatic flexure, transverse; the *P*_heterogeneity_ for right colon vs rectum or rectosigmoid associations was .001 and .0002 for the overall DIS and LIS models, respectively.

The LIS was more strongly, directly associated with CRC risk than was the DIS, particularly among men (Table 3). Among those in the highest relative to the lowest LIS quintile, risk was 38% (95% CI = 30% to 48%) higher overall, and 49% and 22% higher among men and women, respectively. Overall, among those in the highest relative to the lowest LIS quintiles, risk for left- and right-side colon cancers was statistically significantly 59% and 40% higher, respectively, but for rectal cancers it was an estimated nonstatistically significant 13% higher. The estimated colorectal site differences were greater among men than among women.

The joint and combined (cross-classification) associations of the DIS and LIS with CRC risk are presented in Table 4. Overall and among men and women separately, the highest CRC risk was among those in the highest relative to the lowest joint DIS and LIS quintile (83% [95% CI = 68% to 99%] higher overall, twofold higher among men, and 55% higher among women; all *P*_interaction_ < .01).

**Table 4.** Joint and combined associations of the DIS and LIS* with incident CRC overall and by sex; the NIH-AARP, 1995–2011 (N = 453 465)

Population and DIS quintiles^†^	LIS quintiles^†^										
	1		2		3		4		5		
	No. cases	HR (95% CI)	No. cases	HR (95% CI)	No. cases	HR (95% CI)	No. cases	HR (95% CI)	No. cases	HR (95% CI)	*P*_interaction_
All participants											
1	361	1.00 (Referent)	478	1.13 (1.06 to 1.20)	351	1.22 (1.15 to 1.30)	381	1.23 (1.16 to 1.31)	306	1.41 (1.32 to 1.50)	
2	371	1.02 (0.96 to 1.09)	398	1.15 (1.05 to 1.26)	362	1.25 (1.14 to 1.36)	436	1.26 (1.15 to 1.37)	338	1.43 (1.31 to 1.57)	
3	341	1.07 (1.01 to 1.14)	391	1.21 (1.10 to 1.32)	423	1.31 (1.20 to 1.43)	474	1.32 (1.21 to 1.44)	379	1.51 (1.38 to 1.65)	
4	335	1.14 (1.07 to 1.21)	365	1.28 (1.17 to 1.40)	469	1.39 (1.27 to 1.51)	479	1.40 (1.28 to 1.53)	478	1.60 (1.46 to 1.74)	
5	319	1.30 (1.22 to 1.38)	346	1.46 (1.34 to 1.60)	550	1.59 (1.46 to 1.73)	545	1.60 (1.47 to 1.75)	660	1.83 (1.68 to 1.99)	<.001
Men											
1	214	1.00 (Referent)	337	1.15 (1.06 to 1.24)	229	1.30 (1.20 to 1.41)	269	1.27 (1.17 to 1.37)	194	1.52 (1.40 to 1.65)	
2	225	1.04 (0.97 to 1.13)	304	1.20 (1.07 to 1.34)	250	1.36 (1.22 to 1.52)	297	1.33 (1.19 to 1.48)	221	1.59 (1.42 to 1.78)	
3	193	1.08 (1.00 to 1.17)	292	1.24 (1.11 to 1.39)	287	1.41 (1.26 to 1.57)	329	1.37 (1.23 to 1.53)	239	1.64 (1.47 to 1.84)	
4	201	1.12 (1.04 to 1.21)	268	1.29 (1.15 to 1.44)	326	1.46 (1.31 to 1.63)	312	1.42 (1.28 to 1.59)	290	1.71 (1.53 to 1.91)	
5	219	1.32 (1.23 to 1.43)	253	1.52 (1.36 to 1.70)	385	1.72 (1.55 to 1.92)	381	1.68 (1.51 to 1.87)	390	2.01 (1.80 to 2.24)	<.001
Women											
1	147	1.00 (Referent)	141	1.11 (0.99 to 1.25)	122	1.08 (0.97 to 1.21)	112	1.17 (1.05 to 1.30)	112	1.23 (1.11 to 1.37)	
2	146	0.97 (0.87 to 1.09)	94	1.08 (0.92 to 1.27)	112	1.05 (0.90 to 1.23)	139	1.13 (0.97 to 1.32)	117	1.20 (1.03 to 1.39)	
3	148	1.06 (0.95 to 1.18)	99	1.18 (1.01 to 1.39)	136	1.15 (0.99 to 1.34)	145	1.24 (1.07 to 1.44)	140	1.31 (1.13 to 1.52)	
4	134	1.16 (1.04 to 1.29)	97	1.29 (1.10 to 1.51)	143	1.26 (1.09 to 1.46)	167	1.36 (1.17 to 1.58)	188	1.43 (1.24 to 1.66)	
5	100	1.26 (1.13 to 1.40)	93	1.40 (1.20 to 1.64)	165	1.36 (1.18 to 1.58)	164	1.47 (1.27 to 1.70)	270	1.55 (1.34 to 1.79)	.004

* Inflammation scores constructed as described in the text and Table 1; a higher score reflects a higher balance of proinflammatory exposures. CI = confidence interval; CRC = colorectal cancer; DIS = dietary inflammation score; HR = hazards ratio; HRT = hormone replacement therapy; LIS = lifestyle inflammation score; NIH-AARP = National Institutes of Health American Association for Retired Persons Diet and Health Study.

† Covariates in the Cox proportional hazards models were: age at entry (continuous), sex, race (black, white, or other), education (less than high school and high school graduate, some college, or college graduate or higher), marital status (married or nonmarried), heart disease or history of stroke at baseline (yes or no), diabetes mellitus at baseline (yes or no), emphysema at baseline (yes or no), gallstone or gallbladder disease at baseline (yes or no), current HRT use (among women), family history of CRC in a first-degree relative, history of colon polyp, total energy intake (kcal/d), and former smoker (yes or no); history of CRC in a first-degree relative, self-reported heart disease diagnosis, age at entry, and sex were included in the SAS *STRATA* statement.

DIS–CRC and LIS–CRC associations according to selected participant characteristics (Supplementary Table 2, available online) were generally similar across most baseline characteristics. There were no consistent, clear patterns of differences in DIS–CRC associations; however, the LIS associations tended to be stronger among men and among women using hormone replacement therapy.

In sensitivity analyses, as hypothesized, the equally weighted DIS and LIS (Supplementary Table 3, available online) were somewhat more strongly, directly associated with CRC than were the weighted scores (overall, the estimated risks among those in the highest relative to the lowest equal-weight DIS and LIS quintiles were statistically significantly 35% and 55% higher, respectively). The DIS without supplemental micronutrients–CRC associations were negligibly weaker (Supplementary Table 4, available online). The reverse Healthy Eating Index (HEI)–CRC associations were somewhat stronger than those for the DIS but were very similar to those for the equally weighted DIS. The EDIP–CRC associations were not statistically significant and much closer to null than were those for the DIS (Supplementary Table 5, available online). The findings for individual LIS components (Supplementary Table 6, available online) were weaker than those for the LIS. For example, current relative to never smokers had a 29% higher risk for CRC, those with an obese relative to a normal body mass index had a 24% higher risk, heavy relative to nondrinkers had a 23% higher risk, and those who exercised 3 times or more or 1–2 times weekly relative to those who rarely or never exercised had a 15% and 8% lower risk, respectively. Finally, excluding those who died or were diagnosed with CRC less than 2 years from baseline (Supplementary Table 7, available online) negligibly affected our estimated associations.

## Discussion

Our findings suggest that 1) higher pro- to anti-inflammatory balances of either dietary or lifestyle exposures, and especially of both combined, may be associated with higher CRC risk; and 2) these direct associations may be stronger among men and for colon cancers.

Inflammation is strongly, mechanistically linked to colorectal carcinogenesis. First, colorectal carcinogenesis is characterized by progressive increases in the expression of cyclooxygenase-2 (COX-2), which is proinflammatory and protumorigenic (30), and approximately 85% of colorectal adenocarcinomas express COX-2 (7). NSAID use reduced colorectal adenoma recurrence and has been consistently associated with lower CRC risk, likely through COX-2 inhibition (7,10,12,31–35). Second, higher circulating inflammation biomarker concentrations have been associated with CRC risk. For example, in a meta-analysis of 18 nested case-control studies, 12% higher risk for incident CRC for every one-unit increase in baseline log-transformed CRP concentrations was found (36). Finally, individuals with inflammatory bowel diseases have higher CRC risk (37,38).

Risk for colorectal neoplasms is also highly associated with dietary and various lifestyle exposures (13,39). Considerable evidence supports positive associations of obesity, heavy alcohol intake, and smoking with CRC, and inverse physical activity–CRC associations (40–46). Furthermore, dietary patterns characterized by high vegetable, fruit, whole grain, low-fat dairy, fish, poultry, olive oil, and legume intakes are consistently inversely associated with colorectal neoplasms, whereas dietary patterns characterized by high red and processed meat, refined grain, foods with added sugar, potato, and saturated and trans fat intakes are consistently positively associated with colorectal neoplasms (47,48). In the NIH-AARP, multiple previous analyses of dietary patterns, defined using different methods, have consistently been associated with CRC risk (49–54). For example, higher relative to lower HEI-2005 scores (55) were associated with 28% and 20% lower CRC risk among men and women, respectively, and higher relative to lower Mediterranean Diet scores were associated with 28% and 11% lower CRC risk among men and women, respectively (52). Our direct DIS–CRC association was slightly weaker than those for the reverse-direction HEI-2015 and equally weighted DIS. This was hypothesized, because the intent of the DIS is to assess the collective contributions of foods to systemic inflammation, and so it comprises components weighted only according to their estimated contributions to systemic inflammation. Thus, the DIS would not address its components’ other potential independent pro- and anticarcinogenic effects. However, the similarity of our DIS findings to our equally weighted DIS and reverse-direction HEI findings suggest that the strong associations of diet with CRC risk may largely involve diet’s contributions to inflammation (described in Supplementary Table 1, available online).

In our study, the DIS was strongly, directly associated with CRC risk, particularly among men. Other food frequency questionnaire–based dietary inflammation scores, the DII and EDIP, have been used to investigate associations of diet-associated inflammation with CRC risk. A meta-analysis of DII–CRC associations from four prospective cohort studies and five case-control studies yielded an estimated 6% higher CRC risk per one-unit increase in the DII (56). One of the included studies was NIH-AARP (with follow-up until 2006), in which, similar to our DIS, the DII was more strongly directly associated with CRC risk among men than among women (HR = 1.44, 95% CI = 1.29 to 1.61; and HR = 1.12, 95% CI = 0.95 to 1.31, respectively) in the highest relative to the lowest DII quartile (16). An association of the EDIP (which was developed in a subset of the Nurses’ Health Study [NHS] cohort) with CRC was investigated in two prospective cohorts, the NHS (all women) and the Health Professionals Follow-up Study (all men); similar to our DIS findings, but not our null EDIP findings, among those in the highest relative to the lowest EDIP quintile, CRC risk was 44% and 22% higher in the Health Professionals Follow-up Study and NHS, respectively (57).

When conceptualizing the implications of the previous DII–CRC or EDIP–CRC associations, it is important to consider their limitations (see Supplementary Table 8, available online for comparison of DIS, LIS, DII, and EDIP). The DII is primarily nutrient based and thus may not account for other whole food constituents that affect inflammation. Although the EDIP is whole foods based, it was developed using a primarily data-driven approach in a demographically, occupationally homogenous population, so its weights may only be reproducible in certain populations (58,59), thus possibly accounting for the more attenuated EDIP–CRC associations observed in our study. Finally, there are no reported lifestyle-specific inflammation scores. The DIS and LIS were developed to address many of these limitations.

Our study had several strengths. First was the prospective design, the large sample size and number of cases, and the excellent case ascertainment and participant follow-up (24). Second, our findings were robust to multiple sensitivity analyses. Third, strengths of the DIS and LIS include their previous validation via comparing their associations with multiple circulating inflammation biomarkers in three study populations (17). In those studies, the DIS was more strongly, directly associated with the circulating biomarkers than was the DII and EDIP, and the LIS was more strongly, directly associated with the biomarkers than was any diet score. Fourth, the DIS is based primarily on whole foods and the LIS on lifestyle factors, facilitating application to population and clinical recommendations for CRC prevention. Fifth, to our knowledge, this is the first study to prospectively investigate a validated LIS, alone or jointly with a DIS, in association with incident CRC.

Our study also had limitations. First, self-reported dietary, lifestyle, and other covariate data are prone to measurement error; however, given our prospective design, these limitations would be nondifferential and thus likely attenuated our observed associations. Furthermore, our study’s DHQ was validated via calibration with 24-hour food recalls in a subset of the NIH-AARP cohort (19,21), and diet patterns calculated using the DHQ have been consistently associated with CRC (49–52). Second, we had data on NSAID use, which is strongly associated with CRC risk, in only a subset of the cohort; however, among participants with NSAID use data, findings adjusted for or stratified by regular aspirin or other NSAID use were not meaningfully different.

In conclusion, our findings, together with previous literature, suggest that a higher balance of pro- relative to anti-inflammatory diets and lifestyles, alone and especially in combination, may be associated with higher CRC risk. Future investigations of associations of diet- and lifestyle-associated inflammation with CRC incidence and survival using our novel DIS and LIS are warranted.

## Funding

This work was supported by the Intramural Research Program in the Division of Cancer Epidemiology and Genetics, the US National Institutes of Health (NIH), National Cancer Institute (NCI), and the Anne and Wilson P. Franklin Foundation (RMB).

## Notes

The study sponsors played no role in designing the study; the collection, analysis, and interpretation of the data; the writing of the manuscript; and the decision to submit the manuscript for publication.

Doratha Byrd, Suzanne Judd, Dana Flanders, Terry Hartman, Veronika Fedirko, Tanya Agurs-Collins, and Roberd M. Bostick declare no conflicts of interest.

## Supplementary Material

pkaa009_Supplementary_Data
