# Supplementary material for: Associations of Novel Dietary and Lifestyle Inflammation Scores With Incident Colorectal Cancer in the NIH-AARP Diet and Health Study
Source: JNCI Cancer Spectr. 2020 Mar 5;4(3):pkaa009. doi: 10.1093/jncics/pkaa009 (PMC7236782; doi:10.1093/jncics/pkaa009)
Supplement: pkaa009_Supplementary_Data [file pkaa009_supplementary_data.pdf]

Supplementary Methods

Supplementary Methods: Detailed exclusion criteria for NIH-AARP

A total of 566,398 respondents completed the baseline questionnaire and provided informed consent. We excluded from analysis participants who responded by proxy (N = 15,760), had a self-reported (N = 49,318) or Surveillance, Epidemiology, and End Results (SEER) registry ascertained (N = 2,007) CRC or other cancer diagnosis before study entry, had self-reported end-stage renal disease (N = 997), had death-only ascertainment of CRC (N = 912) or other cancers (N = 4,017), had implausible total energy intakes (< 500 or > 6,000 kcal/day; N = 6,240) estimated from the Diet History Questionnaire (DHQ), skipped > 15% of the DHQ questions (N = 4,346), had missing self-reported height/weight (N = 11,009) or height/weight > 3 interquartile ranges outside the 75_{th} and 25_{th} percentiles (N = 818; (1)), or had other missing lifestyle questions (N = 7,509). The final analytic sample size was 453,465.

Supplementary Methods: Outcome Ascertainment

We defined incident CRC cases according to *International Classification of Diseases for Oncology* codes C180-C189, C199, C209, and C260.

Supplementary Methods: Description of the DIS and LIS

Briefly, the 19 and four components of the DIS and LIS, respectively, were determined and grouped *a priori*, based on prior literature, biological plausibility, and ease of re-creating them using a variety of FFQs used in major epidemiologic studies, using Block 98 FFQ (2) and lifestyle questionnaire responses (Table S1) in a diverse subset (N = 639) of participants in the previously-described REasons for Geographic and Racial Differences in Stroke Study

(REGARDS) cohort (3,4). REGARDS is a national, on-going prospective cohort study that recruited 30,239 male, female, white, and black participants ≥ 45 years old from the 48 contiguous states of the United States, intiated January 2003 – October 2007, with oversampling of black and Southeastern US residents. We excluded from the analytic sample participants with circulating CRP concentrations > 10 mg/dL, extreme outlying values for other inflammation biomarker concentrations, implausible energy intakes (< 500 or > 6,000 kcal/day), > 10% missing FFQ items, ≥ 2 comorbidities, end-stage renal disease, and age ≥ 75 years.

Weights for the DIS and LIS components were calculated in REGARDS based on their multivariable-adjusted strengths of associations with an inflammation biomarker score. To create the biomarker score, for each participant, plasma inflammation biomarker concentrations were transformed by the natural logarithm, normalized, and then summed (high-sensitivity C-reactive protein (hsCRP), interleukin (IL)-6, IL-8, and IL-10 [the latter with a negative sign]). Next, each DIS component (all continuous) was standardized, by sex, to a mean of 0 and standard deviation of 1.0; indicator variables were created for the LIS components (all categorical). Then, associations of the DIS and LIS components with the biomarker score were estimated using multivariable linear regression. The β-coefficients from the score component-biomarker score associations were taken as the components' weights. When the DIS scoring procedures and weights were applied in three different external populations in which different Willett FFQ versions were used, the DIS was more strongly directly associated with circulating biomarkers of inflammation than was the DII or EDIP (5). The estimated associations of the DIS and LIS with inflammation biomarkers were similar across sex and race (5).

Supplementary Methods: Calculating the DIS and LIS in the NIH-AARP Diet and Health Study

In NIH-AARP, we standardized each food group and the supplement score, by sex, to a mean of 0 and standard deviation of 1.0 based on the baseline distribution among all participants; i.e.,

$$\frac{X - \mu}{\sigma}$$

where μ denotes the study population mean, σ denotes the study population standard deviation, and X denotes the participant's intake.

For the LIS, baseline smoking status was categorized as 'current' or 'former and never'. Baseline body mass index (BMI) was categorized as normal (18.5 – 24.99 kg/m_2), overweight (25 – 29.99 kg/m_2), or obese (BMI ≥ 30 kg/m_2). Baseline heavy alcohol consumption for men and women was defined as > 2 or > 1 drinks/day, respectively; moderate consumption was defined as individuals consuming alcohol in less than these amounts. For physical activity, we categorized participants as those who did not or rarely exercised, exercised 1 – 2 times/week, or exercised ≥ 3 times/wk.

Supplementary Methods: Statistical Analyses

Prior to conducting the Cox proportional hazards regression, we assessed the proportional hazards assumption by calculating Martingale and Schoenfeld residuals, testing time-dependent covariates, and inspecting log(-log) survival curves for each variable in the model. Variables that violated the proportional hazards assumptions were included in the SAS *STRATA* statement in all models and are listed in the table footnotes. We ruled out multicollinearity considering a condition index ≥ 30 and a variance decomposition proportion ≥ 0.5 as evidence of multicollinearity.

Potential confounders were based on biological plausibility, previous literature on their associations with CRC, inflammation, or dietary and lifestyle exposures, and causal diagrams. Covariates considered for all models included age, sex, race, education, marital status, comorbidities (self-reported gallbladder disease, heart disease, emphysema, or diabetes mellitus), hormone replacement therapy use (among women), family history of CRC in a first degree relative, self-reported history of colon polyps, and total energy intake. Covariates considered for the DIS models also included individual covariates for smoking status, BMI, alcohol intake, and physical activity since they are strong CRC risk factors with hypothesized contributions to colorectal carcinogenesis through inflammation plus other independent pro- or anti-carcinogenic pathways. Covariates considered for the LIS models included former smoking status since the LIS only includes current smoking at baseline as a component, and former tobacco smoking is also associated with CRC risk. LIS models also included the equally-weighted DIS, since the 19 DIS components individually are weak CRC risk factors, to account for the components inflammation plus other colorectal carcinogenic-related effects, and to reduce model size (including the score components individually or collectively in the equally-weighted dietary inflammation score yielded no substantial differences in our estimated LIS-CRC association estimated HRs). We also considered adjustment for regular aspirin and other NSAID use in the subset of the cohort that completed RFQs 6 months from baseline, and adjustment for colonoscopy screening over follow-up in the subset that completed follow-up questionnaires from 2004 – 2005; however, adjustment for these covariates did not materially change the estimated DIS/LIS-CRC associations.

We also investigated potential effect modification by conducting separate analyses for the DIS and LIS within categories of age (< / ≥ 65 years), sex and hormone replacement therapy use (among women), race (white, black, or other), baseline comorbidity (yes/no), family history of CRC in a first degree relative (yes/no), and for the DIS, baseline smoking status (never, former, or current), BMI (normal, overweight, or obese), baseline alcohol intake (non-drinker, moderate drinker, or heavy drinker), and baseline physical activity (exercises never or rarely, 1 – 3 times/week, ≥ 3 times/week). In a subset of the cohort that completed RFQs 6 months from their baseline questionnaire, we conducted analyses within strata of regular aspirin or other NSAID use (≥ once/week). In the subset that completed follow-up questionnaires from 2004 – 2005, we excluded participants who were diagnosed with CRC or were otherwise censored prior to 2004, and conducted analyses within strata of time since their last colonoscopy during follow up (never, < 5 years ago, ≥ 5 years ago). We assessed effect modification by comparing the stratum-specific estimates and by calculating Wald test p-values for model interaction terms.

To test statistically for heterogeneity by CRC site, we conducted a case-only analysis using multivariable logistic regression. The dependent variable was the CRC subtype (i.e., colon, left colon, right colon, and rectum/rectosigmoid cancer) with rectum/rectosigmoid cancer as the referent group. The independent variables were the DIS and LIS modeled continuously, with the same covariates as in the Cox proportional hazards models plus person-time (years of follow-up). We took the *P*-value for the continuous DIS and LIS to be the $P_{heterogeneity}$.

Supplementary Methods: Sensitivity Analyses, Expanded with Rationales

To assess the sensitivity of the associations to various considerations, we repeated the analyses with the following variations. First, to explore potential differences in inflammation-related vs. total contributions of the aggregated score components to risk, we constructed equally-weighted DIS and LIS versions by assigning positive or negative equal weights to dietary/lifestyle components we hypothesized *a priori* to be pro-inflammatory or anti-inflammatory, respectively. Second, to rule out a substantial influence of the the supplement score component in our DIS, we calculated a DIS without Supplementary micronutrients and assessed its association with CRC. Third, we calculated a reverse-direction Healthy Eating Index-2015 (reverse HEI-2015; i.e., so a higher score would be higher risk) (6), and the empirical dietary inflammatory pattern (EDIP), as described by Tabung et al. (7), and investigated their associations with CRC. Fourth, we investigated associations of each individual lifestyle component with CRC. Fifth, we excluded individuals who died or were diagnosed with CRC within two years from baseline to rule out spurious effects on risk estimates by participants with an undiagnosed CRC or morbid illness that may have affected their long-term diets or lifestyles at the time of questionnaire completion.

References for Supplementary Methods

1. Adams KF, Leitzmann MF, Albanes D, Kipnis V, Mouw T, Hollenbeck A, Schatzkin A. Body mass and colorectal cancer risk in the NIH-AARP cohort. Am J Epidemiol [Internet]. 2007 Apr 9 [cited 2018 Aug 24];166(1):36–45. Available from: https://academic.oup.com/aje/article-abstract/166/1/36/135568
2. Boucher B, Cotterchio M, Kreiger N, Nadalin V, Block T, Block G. Validity and reliability of the Block98 food-frequency questionnaire in a sample of Canadian women. Public Health

Nutr [Internet]. 2006 Feb 2 [cited 2018 Jan 10];9(1):84–93. Available from: https://www.cambridge.org/core/services/aop-cambridge-core/content/view/96E0CA3E5196B78C1A8E804D0FD38D4B/S1368980006000139a.pdf/validity_and_reliability_of_the_block98_foodfrequency_questionnaire_in_a_sample_of_canadian_women.pdf

3. Howard VJ, Cushman M, Pulley L, Gomez CR, Go RC, Prineas RJ, Graham A, Moy CS, Howard G. The Reasons for Geographic and Racial Differences in Stroke Study: objectives and design. Neuroepidemiology [Internet]. 2005 [cited 2017 Mar 12];25(3):135–43. Available from: www.karger.com
4. Olson NC, Cushman M, Lutsey PL, McClure LA, Judd S, Tracy RP, Folsom AR, Zakai NA. Inflammation markers and incident venous thromboembolism: the REasons for Geographic And Racial Differences in Stroke (REGARDS) cohort. J Thromb Haemost [Internet]. 2014 Dec [cited 2018 Jun 26];12(12):1993–2001. Available from: https://www-ncbi-nlm-nih-gov.proxy.library.emory.edu/pmc/articles/PMC4643856/pdf/nihms726632.pdf
5. Byrd DA, Judd SE, Flanders WD, Hartman TJ, Fedirko V, Bostick RM. Development and validation of novel dietary and lifestyle inflammation scores. J Nutr [Internet]. 2019 Aug 2;[Epub ahea. Available from: https://academic.oup.com/jn/advance-article/doi/10.1093/jn/nxz165/5542977
6. Krebs-Smith SM, Pannucci TE, Subar AF, Kirkpatrick SI, Lerman JL, Tooze JA, Wilson MM, Reedy J. Update of the Healthy Eating Index: HEI-2015. J Acad Nutr Diet [Internet]. 2018 Sep 1 [cited 2018 Nov 12];118(9):1591–602. Available from: https://www-sciencedirect-com.proxy.library.emory.edu/science/article/pii/S2212267218308384

7. Tabung FK, Smith-Warner SA, Chavarro JE, Wu K, Fuchs CS, Hu FB, Chan AT, Willett WC, Giovannucci EL. Development and Validation of an Empirical Dietary Inflammatory Index. J Nutr [Internet]. 2016 Aug 1 [cited 2017 Feb 1];146(8):1560–70. Available from: https://academic.oup.com/jn/article-abstract/146/8/1560/4630463

Supplementary Tables

Supplementary Table 1. Components of the dietary (DIS) and lifestyle (LIS) inflammation scores and their rationales for inclusion

Components	Rationales for inclusion
LIS components*	
Overweight BMI	Adipose tissue synthesizes and releases pro-inflammatory adipokines, such as PA-1 and TNF-α (1,2)
Obese BMI	Mechanisms similar to those described above
Heavy drinker	Heavy alcohol intake results in oxidative stress via oxidation of ethanol to acetaldehyde (3,4)
Moderate drinker	A metabolite of ethanol is acetate, which can acutely lower pro-inflammatory free fatty acid concentrations; moderate alcohol intake increases serum adiponectin concentrations (an anti-inflammatory inflammation biomarker) (5) and inhibits IL-6 production and activity (6)
Moderately physically active	Physical activity improves systemic plasma antioxidant capacity (increases adaptive responses to oxidative stress), increases concentrations of anti-inflammatory cytokines, and lowers vascular wall inflammation (2,7)
Heavily physically active	Mechanisms similar to those described above
Current smoker	Toxins injure tissues, upregulating cytokines and acute phase reactants (8)
DIS components*	
Leafy greens and cruciferous vegetables	Contain variety of potent antioxidants (e.g., β-carotene, folacin, magnesium, calcium, glucosinolates, isothiocyanates, lutein, and indoles); contain flavonoids and polyphenols, which activate the transcription factor, Nrf2, which plays a key role in cellular protection against oxidative stress and inflammation (9,10, 19,11–18)
Tomatoes	Contain β-carotene, vitamin C, and lycopene, the latter of which is a potent singlet oxygen quencher and one of the most powerful antioxidants among the natural carotenoids (20–23)
Apples and berries	Contain flavonoids (e.g., anthocyanins, quercetin, and phenolic acids) that suppress pro-inflammatory cytokine production and are powerful antioxidants; potentially increase postprandial plasma antioxidant capacity (24–26)
Deep yellow or orange vegetables and fruit	Contain pro-vitamin A carotenoids (e.g., β-carotene and α-carotene), which have a conjugated double-bond structure making them strong antioxidants (27)
Other fruits and real fruit juices	Contain antioxidants (e.g., flavonoids, such as hesperidin, naringenin, neohesperidin, limonene, vitamin C, β-cryptoxanthin, plant sterols, salicylates, naringin, nobelitin, and narirutin) with similar mechanisms to those described above (13,28–35)
Other vegetables	Contain antioxidants and polyphenols with similar mechanisms to those described above

Legumes	Contain folacin, iron, isoflavones, protein, vitamin B6, and have a high antioxidant capacity; rich in fiber, which is associated with beneficial alterations to the gut microbiota, reducing immune response in the gut (12,36,37)
Fish	Contain Ω-3 fatty acids, which compete with pro-inflammatory Ω-6 fatty acids by synthesizing eicosanoids and suppress the capacity of monocytes to synthesize IL-1β and TNF-α (38–40)
Poultry	Inversely associated with inflammation markers (41); contain low amounts of saturated fat (42); contain l-arginine, which improves endothelium-dependent dilation (precursor of the endogenous vasodilator nitric oxide) and decreases platelet aggregation and monocyte adhesion (12)
Red and organ meats	Contain heme iron, which increases the bioavailability of iron, which in turn increases oxidative stress; contain Ω-6 fatty acids, which increase oxidative stress through free radical production and are converted to arachidonic acid which stimulates expression of IL-1β and TNF-α in monocytes, and IL-6 and IL-8 in endothelial cells (43–45); contain saturated fats that mimic lipopolysaccharide, a pro-inflammatory stimulant, in the gut, and increase cytotoxic, pro-oxidant, and pro-inflammatory bile acids in the colon (43,46)
Processed meats	Contain heme iron, higher saturated fat contents, Ω-6 fatty acids (see above), and additives, such as nitrites, with suspected pro-inflammatory properties (41,47)
Added sugars	Sparse in nutrients; induce postprandial hyperglycemia, which act as stressful stimuli through subsequent repeated mild postprandial hypoglycemia (48) and reduce nitric oxide availability (plays role in regulation of inflammatory response (49)); elevate pro-inflammatory free fatty acid levels (40); produce oxidative stress through oxidation of membrane lipids, proteins, lipoproteins, and DNA (50)
High-fat dairy	Contains calcium, which binds bile acids and free fatty acids, decreasing oxidative damage in the gut; dairy fat contains fatty acids with potential inflammation-reducing properties, such as CLA, cis- and trans-palmitoleic acid, butyric acid, phytanic acid, and alpha-linolenic acid (51–53)
Low-fat dairy	Similar mechanisms to high-fat dairy (see above), with lower fat content
Coffee and tea	Tea contains flavonoids and antioxidants (e.g., epicatechin and quercetin) (54); coffee contains phytochemicals and antioxidants, such as javamide; both coffee and tea contain varying amounts of caffeine which inhibit secretion of IL-1β induced by adenine and N4-acetylcytidine (36,55)
Nuts	Contain Ω-3 fatty acids (38, 40,56,57) and l-arginine (12) (mechanisms similar to those described above in ‘Fish’ and ‘Poultry’)
Other fats	Contain Ω-6 fatty acids and saturated fats (see ‘red and organ meats’ above)

Refined grains and starchy vegetables	Some processed grains contain emulsifiers, which potentially break down mucin in the gut leading to inflammation (58); and induce hyperglycemia (mechanisms described similar to those described above in 'Added Sugars')
Supplement score†	Comprises micro-nutrients, minerals, and vitamins solely from supplement intakes, some with similar mechanisms to those described above (e.g., iron as pro-oxidant, vitamins A, C, and E as antioxidants)

Abbreviations: BMI, body mass index; CLA, conjugated linoleic acids; DIS, dietary inflammation score; hsCRP, high-sensitivity C-reactive protein; IL, interleukin; LIS, lifestyle inflammation score; METs, metabolic equivalents of task; Nrf2, Nuclear factor-erythroid 2 (NF-E2)-related factor 2; NSAID, nonsteroidal anti-inflammatory drug; PA1, plasminogen activator inhibitor–1; TNF, tumor necrosis factor

* Weights are β coefficients from multivariable linear regression models conducted in a subset of the REGARDS cohort study (n = 639), and represent the average change in an inflammation biomarker score (sum of z-scores for circulating hsCRP, IL-6, IL-8, and IL-10 [the latter with a negative sign]) concentrations per one standard deviation increase in a dietary component or the presence of lifestyle component. Covariates in the final model to develop the weights included: age, sex, race (Black or White), education (high school graduate or less vs. some college or more), region (stroke belt, stroke buckle, or other region in the US), a comorbidity score (comprises a history of cancer, heart disease, diabetes mellitus, or chronic kidney disease), hormone replacement therapy (among women), total energy intake (kcal/day), season of baseline interview (Spring, Summer, Fall, or Winter), and regular use of aspirin, other non-steroidal anti-inflammatory drugs or lipid-lowering medications (≥ twice/wk); and all the dietary/lifestyle components in the DIS and LIS. For the NIH-AARP study, all dietary components were standardized based on their sex-specific distributions in the analytic cohort at baseline, and all lifestyle components were dummy variables.

† All vitamin and mineral supplement intakes measured (from multivitamin/mineral and individual supplements) were ranked into quantiles of intake and assigned a value of 0 (low or no intake), 1, or 2 (highest intake) for hypothesized anti-inflammatory supplements (e.g., vitamin E), and 0 (low or no intake), -1, or -2 (highest intake) for hypothesized pro-inflammatory supplements (e.g., iron)

Supplementary Table 2. Associations of the dietary (DIS) and lifestyle (LIS) inflammation scores* with incident colorectal cancer by selected characteristics; the NIH-AARP Diet and Health Study (NIH-AARP; n = 453,465), 1995 – 2011

Characteristics	No. cases	Quintile 1	Quintile 2	Quintile 3	Quintile 4	Quintile 5	P_{trend}	$P_{interaction}$ †
DIS, ‡								
Age, y								
< 65	5,567	1.00 (Referent)	1.01 (0.92, 1.10)	1.04 (0.95, 1.14)	1.06 (0.97, 1.16)	1.24 (1.14, 1.36)	<0.001	
≥ 65	4,769	1.00 (Referent)	1.01 (0.92, 1.12)	1.07 (0.98, 1.18)	1.17 (1.07, 1.29)	1.29 (1.18, 1.42)	<0.001	0.64
HRT use and sex								
Men	6,905	1.00 (Referent)	1.04 (0.96, 1.12)	1.07 (0.99, 1.15)	1.10 (1.02, 1.19)	1.29 (1.19, 1.39)	<0.001	
Women on HRT	1,197	1.00 (Referent)	0.93 (0.78, 1.12)	1.01 (0.85, 1.22)	1.03 (0.85, 1.24)	1.25 (1.03, 1.51)	0.02	
Women not on HRT	2,234	1.00 (Referent)	0.98 (0.85, 1.13)	1.05 (0.91, 1.21)	1.19 (1.04, 1.37)	1.21 (1.05, 1.39)	0.001	0.33
Race								
White	9,546	1.00 (Referent)	1.01 (0.94, 1.08)	1.05 (0.98, 1.12)	1.10 (1.03, 1.17)	1.26 (1.18, 1.35)	<0.001	
Black	396	1.00 (Referent)	1.11 (0.70, 1.76)	1.23 (0.78, 1.92)	1.44 (0.94, 2.19)	1.31 (0.87, 1.97)	0.14	
Other	394	1.00 (Referent)	0.93 (0.63, 1.38)	1.00 (0.69, 1.45)	1.36 (0.95, 1.96)	1.05 (0.72, 1.52)	0.34	0.28
Comorbidity[‖]								
No	7,047	1.00 (Referent)	1.05 (0.97, 1.13)	1.08 (1.00, 1.16)	1.14 (1.06, 1.24)	1.28 (1.19, 1.39)	<0.001	
Yes	3,289	1.00 (Referent)	0.94 (0.83, 1.05)	1.02 (0.91, 1.15)	1.05 (0.94, 1.18)	1.21 (1.08, 1.36)	<0.001	0.36
Family history of CRC[¶]								
No	8,868	1.00 (Referent)	1.01 (0.94, 1.08)	1.06 (0.99, 1.13)	1.12 (1.05, 1.20)	1.26 (1.18, 1.35)	<0.001	
Yes	1,019	1.00 (Referent)	1.00 (0.82, 1.23)	0.95 (0.77, 1.16)	1.00 (0.81, 1.23)	1.22 (0.99, 1.50)	0.08	0.78
Tobacco use								
Non-smoker	3,251	1.00 (Referent)	0.96 (0.86, 1.08)	1.03 (0.92, 1.15)	1.07 (0.96, 1.20)	1.20 (1.07, 1.34)	<0.001	
Former smoker	5,792	1.00 (Referent)	1.06 (0.98, 1.16)	1.09 (1.00, 1.19)	1.16 (1.07, 1.27)	1.37 (1.26, 1.49)	<0.001	
Current smoker	1,293	1.00 (Referent)	0.86 (0.68, 1.09)	0.93 (0.74, 1.16)	0.90 (0.73, 1.11)	0.98 (0.80, 1.19)	0.70	0.28
BMI, kg/m^2								
18.5 – 24.99	3,181	1.00 (Referent)	1.07 (0.95, 1.19)	0.99 (0.88, 1.11)	1.11 (0.99, 1.25)	1.29 (1.15, 1.45)	<0.001	
25 – 29.99	4,619	1.00 (Referent)	0.92 (0.84, 1.02)	1.07 (0.98, 1.18)	1.13 (1.03, 1.24)	1.25 (1.14, 1.37)	<0.001	
≥ 30	2,515	1.00 (Referent)	1.13 (0.98, 1.29)	1.11 (0.97, 1.27)	1.09 (0.95, 1.25)	1.26 (1.11, 1.44)	0.002	0.26
Alcohol use[#]								
Non-drinker	2,408	1.00 (Referent)	1.07 (0.92, 1.24)	1.00 (0.87, 1.16)	1.15 (1.00, 1.33)	1.30 (1.13, 1.49)	<0.001	
Moderate drinker	6,907	1.00 (Referent)	1.01 (0.93, 1.09)	1.08 (1.00, 1.17)	1.08 (1.00, 1.17)	1.23 (1.14, 1.34)	<0.001	
Heavy drinker	1,021	1.00 (Referent)	1.00 (0.80, 1.25)	1.00 (0.80, 1.25)	1.30 (1.05, 1.61)	1.48 (1.20, 1.83)	<0.001	0.21
Physical activity								
Rarely or never exercises	2,069	1.00 (Referent)	0.82 (0.68, 0.98)	0.93 (0.79, 1.11)	0.96 (0.81, 1.13)	1.09 (0.93, 1.28)	0.01	
Exercises 1 – 2 times/wk	3,717	1.00 (Referent)	1.01 (0.90, 1.14)	0.99 (0.89, 1.11)	1.08 (0.97, 1.21)	1.20 (1.07, 1.34)	<0.001	
Exercises ≥ 3 times/wk	4,550	1.00 (Referent)	1.06 (0.97, 1.16)	1.11 (1.01, 1.22)	1.17 (1.06, 1.28)	1.35 (1.22, 1.48)	<0.001	0.27

Take aspirin ≥ once/wk**								
No	3,913	1.00 (Referent)	1.06 (0.95, 1.18)	1.07 (0.96, 1.20)	1.23 (1.11, 1.37)	1.34 (1.21, 1.49)	<0.001	
Yes	2,541	1.00 (Referent)	1.06 (0.93, 1.20)	1.19 (1.05, 1.35)	1.12 (0.98, 1.28)	1.35 (1.18, 1.54)	<0.001	0.27
Take NSAID ≥ once/wk**								
No	5,238	1.00 (Referent)	1.04 (0.95, 1.14)	1.09 (0.99, 1.19)	1.18 (1.08, 1.29)	1.35 (1.23, 1.48)	<0.001	
Yes	1,204	1.00 (Referent)	1.07 (0.88, 1.31)	1.30 (1.08, 1.58)	1.27 (1.04, 1.54)	1.30 (1.06, 1.59)	0.003	0.18
Had a colonoscopy††								
Never had one	920	1.00 (Referent)	1.15 (0.90, 1.46)	1.18 (0.93, 1.50)	1.24 (0.98, 1.56)	1.23 (0.98, 1.56)	0.08	
< 5 years ago	3,713	1.00 (Referent)	1.02 (0.92, 1.14)	1.04 (0.94, 1.16)	1.17 (1.05, 1.30)	1.32 (1.18, 1.47)	<0.001	
≥ 5 years ago	485	1.00 (Referent)	0.75 (0.54, 1.03)	0.85 (0.62, 1.17)	1.07 (0.78, 1.45)	1.43 (1.05, 1.93)	0.003	0.24
LIS, ‡‡								
Age, y								
< 65	5,567	1.00 (Referent)	1.21 (1.10, 1.33)	1.29 (1.18, 1.41)	1.34 (1.22, 1.46)	1.50 (1.37, 1.64)	<0.001	
≥ 65	4,769	1.00 (Referent)	1.05 (0.96, 1.15)	1.14 (1.04, 1.25)	1.11 (1.01, 1.22)	1.28 (1.16, 1.41)	<0.001	0.07
HRT use and sex								
Men	6,905	1.00 (Referent)	1.15 (1.06, 1.24)	1.29 (1.19, 1.39)	1.26 (1.16, 1.36)	1.49 (1.37, 1.62)	<0.001	
Women on HRT	1,197	1.00 (Referent)	1.11 (0.92, 1.34)	1.21 (1.03, 1.44)	1.40 (1.17, 1.67)	1.39 (1.16, 1.67)	<0.001	
Women not on HRT	2,234	1.00 (Referent)	1.10 (0.95, 1.27)	0.99 (0.86, 1.14)	1.04 (0.91, 1.19)	1.13 (0.99, 1.29)	0.23	0.001
Race								
White	9,546	1.00 (Referent)	1.13 (1.06, 1.21)	1.22 (1.15, 1.31)	1.24 (1.16, 1.32)	1.40 (1.31, 1.50)	<0.001	
Black	396	1.00 (Referent)	1.10 (0.73, 1.65)	0.99 (0.66, 1.50)	1.05 (0.71, 1.53)	1.22 (0.84, 1.78)	0.25	
Other	394	1.00 (Referent)	0.96 (0.70, 1.31)	1.11 (0.82, 1.50)	0.95 (0.69, 1.32)	1.23 (0.88, 1.71)	0.39	0.70
Comorbidity||								
No	7,047	1.00 (Referent)	1.15 (1.06, 1.24)	1.21 (1.13, 1.31)	1.25 (1.16, 1.35)	1.44 (1.33, 1.56)	<0.001	
Yes	3,289	1.00 (Referent)	1.05 (0.93, 1.20)	1.19 (1.05, 1.35)	1.13 (1.00, 1.27)	1.26 (1.12, 1.42)	<0.001	0.18
Family history of CRC¶								
No	8,868	1.00 (Referent)	1.12 (1.05, 1.21)	1.19 (1.11, 1.28)	1.21 (1.13, 1.29)	1.38 (1.29, 1.49)	<0.001	
Yes	1,019	1.00 (Referent)	1.03 (0.83, 1.26)	1.22 (1.00, 1.49)	1.19 (0.98, 1.45)	1.30 (1.05, 1.60)	<0.001	0.43
Take aspirin ≥ once/wk**								
No	3,913	1.00 (Referent)	1.07 (0.97, 1.19)	1.19 (1.08, 1.32)	1.17 (1.05, 1.29)	1.29 (1.16, 1.43)	<0.001	
Yes	2,541	1.00 (Referent)	1.23 (1.08, 1.40)	1.32 (1.16, 1.51)	1.34 (1.18, 1.53)	1.42 (1.24, 1.63)	<0.001	0.40
Takes NSAID ≥ once/wk**								
No	5,238	1.00 (Referent)	1.11 (1.01, 1.21)	1.22 (1.12, 1.33)	1.24 (1.13, 1.35)	1.38 (1.26, 1.51)	<0.001	
Yes	1,204	1.00 (Referent)	1.24 (1.02, 1.51)	1.32 (1.09, 1.60)	1.26 (1.04, 1.52)	1.29 (1.06, 1.57)	<0.001	0.32
Had a colonoscopy††								
Never had one	920	1.00 (Referent)	1.22 (0.97, 1.53)	1.36 (1.09, 1.70)	1.42 (1.15, 1.76)	1.35 (1.07, 1.70)	<0.001	

< 5 years ago	3,713	1.00 (Referent)	1.18 (1.06, 1.30)	1.25 (1.13, 1.39)	1.26 (1.13, 1.39)	1.35 (1.21, 1.51)	<0.001	
≥ 5 years ago	485	1.00 (Referent)	1.06 (0.80, 1.41)	1.16 (0.88, 1.52)	1.08 (0.81, 1.43)	1.13 (0.83, 1.54)	0.44	0.68

Abbreviations: BMI, body mass index; CI, confidence interval; CRC, colorectal cancer; DIS, dietary inflammation score; HEI, Healthy Eating Index; HR, hazard ratio; HRT, hormone replacement therapy; LIS, lifestyle inflammation score; NSAID, non-steroidal anti-inflammatory drug; NIH-AARP, National Institutes of Health-American Association for Retired Persons Diet and Health Study

* Inflammation scores constructed as described in the text and Table 1; a higher score reflects a higher balance of pro-inflammatory exposures

† From interaction term in the full Cox proportional hazards regression model, calculated using the Wald test

‡ Covariates in the DIS Cox proportional hazards models were: age at entry (continuous), sex, race (Black, White, or other), education (less than high school and high school graduate, some college, or college graduate or higher), marital status (married or non-married), heart disease or history of stroke at baseline (yes/no), diabetes mellitus at baseline (yes/no), emphysema at baseline (yes/no), gallstone or gallbladder disease at baseline (yes/no), current hormone replacement therapy use (among women), family history of colorectal cancer in a first degree relative, history of colon polyp, smoking (current, former, or never), body mass index (in kg/m^2; continuous), alcohol intake (non-drinker, moderate-drinker, or heavy-drinker), physical activity level (exercises not at all or rarely, 1 – 2, or ≥ 3 times/wk), and total energy intake (kcal/day); history of CRC in a first degree relative, self-reported heart disease diagnosis, age at entry, sex, and BMI were included in the SAS *STRATA* statement

|| Self-reported heart disease, diabetes mellitus, gallstone or gallbladder disease, or emphysema at baseline

¶ In a first degree relative

Heavy drinker defined as > 1 drink/day for women and > 2 drinks/day drinks for men; moderate drinker defined as 1 drink/day for women, 1 – 2 drinks/day for men

** Aspirin/other NSAID use was ascertained in a subset of the baseline cohort that completed follow-up and risk factor questionnaires (N = 284,211 and N = 283,295, respectively)

†† Colonoscopy history was assessed in remaining baseline cohort members from 2004 – 2005; CRC cases diagnosed prior to 01/01/2004 were excluded from colonoscopy history stratification

‡‡ Covariates in the LIS Cox proportional hazards models were: age at entry (continuous), sex, race (Black, White, or other), education (less than high school and high school graduate, some college, or college graduate or higher), marital status (married or non-married), heart disease or history of stroke at baseline (yes/no), diabetes mellitus at baseline (yes/no), emphysema at baseline (yes/no), gallstone or gallbladder disease at baseline (yes/no), current hormone replacement therapy use (among women), family history of colorectal cancer in a first degree relative, history of colon polyp, total energy intake (kcal/day), former smoker (yes/no), and the equally-weighted DIS; history of CRC in a first degree relative, self-reported heart disease diagnosis, age at entry, and sex were included in the SAS *STRATA* statement

Supplementary Table 3. Associations of the equally-weighted DIS* and LIS* with incident colorectal cancer in the NIH-AARP Diet and Health Study (NIH-AARP; n = 453,465), 1995 – 2011

	Overall		Men		Women	
	DIS-equal weight†	LIS-equal weight‡	DIS-equal weight†	LIS-equal weight‡	DIS-equal weight†	LIS-equal weight‡
Quintiles	Adjusted HR (95% CI)	Adjusted HR (95% CI)	Adjusted HR (95% CI)	Adjusted HR (95% CI)	Adjusted HR (95% CI)	Adjusted HR (95% CI)
1	1.00 (Referent)	1.00 (Referent)	1.00 (Referent)	1.00 (Referent)	1.00 (Referent)	1.00 (Referent)
2	1.04 (0.98, 1.11)	1.17 (1.09, 1.26)	1.05 (0.97, 1.14)	1.17 (1.07, 1.28)	1.02 (0.91, 1.14)	1.19 (1.04, 1.35)
3	1.14 (1.07, 1.22)	1.24 (1.15, 1.34)	1.17 (1.08, 1.27)	1.27 (1.16, 1.39)	1.08 (0.96, 1.21)	1.19 (1.05, 1.36)
4	1.19 (1.11, 1.27)	1.36 (1.26, 1.47)	1.19 (1.10, 1.29)	1.40 (1.27, 1.54)	1.17 (1.04, 1.31)	1.28 (1.13, 1.47)
5	1.35 (1.26, 1.44)	1.55 (1.43, 1.68)	1.38 (1.27, 1.49)	1.64 (1.48, 1.82)	1.28 (1.14, 1.43)	1.40 (1.23, 1.61)

Abbreviations: BMI, body mass index; CI, confidence interval; CRC, colorectal cancer; DIS, dietary inflammation score; HR, hazard ratio; LIS, lifestyle inflammation score; NIH-AARP, National Institutes of Health-American Association for Retired Persons Diet and Health Study

* Dietary and lifestyle components of the equally-weighted inflammation scores are the same as those in the weighted scores (see text and Table 1); weights for all dietary and lifestyle components were equally assigned *a priori* (all in the same direction as the weights in Table 1); higher scores indicate a higher balance of pro- versus anti-inflammatory exposures

† Covariates in the DIS-equal weight Cox proportional hazards models were: age at entry (continuous), sex, race (Black, White, or other), education (less than high school and high school graduate, some college, or college graduate or higher), marital status (married or non-married), heart disease or history of stroke at baseline (yes/no), diabetes mellitus at baseline (yes/no), emphysema at baseline (yes/no), gallstone or gallbladder disease at baseline (yes/no), current hormone replacement therapy use (among women), family history of colorectal cancer in a first degree relative, history of colon polyp, smoking (current, former, or never), body mass index (in kg/m^2; continuous), alcohol intake (non-drinker, moderate-drinker, or heavy-drinker), physical activity level (exercises not at all or rarely, 1 – 2, or ≥ 3 times/wk), and total energy intake (kcal/day); history of CRC in a first degree relative, self-reported heart disease diagnosis, age at entry, sex, and BMI were included in the SAS *STRATA* statement

‡ Covariates in the LIS-weight Cox proportional hazards models were: age at entry (continuous), sex, race (Black, White, or other), education (less than high school and high school graduate, some college, or college graduate or higher), marital status (married or non-married), heart disease or history of stroke at baseline (yes/no), diabetes mellitus at baseline (yes/no), emphysema at baseline (yes/no), gallstone or gallbladder disease at baseline (yes/no), current hormone replacement therapy use (among women), family history of colorectal cancer in a first degree relative, history of colon polyp, total energy intake (kcal/day), former smoker (yes/no), and the equally-weighted DIS; history of CRC in a first degree relative, self-reported heart disease diagnosis, age at entry, and sex were included in the SAS *STRATA* statement

Supplementary Table 4. Associations of the DIS* without Supplemental micronutrients with incident colorectal cancer in the NIH-AARP Diet and Health Study (NIH-AARP; n = 453,465), 1995 – 2011

Quintiles	Overall	Men	Women
	Adjusted HR† (95% CI)	Adjusted HR† (95% CI)	Adjusted HR† (95% CI)
1	1.00 (Referent)	1.00 (Referent)	1.00 (Referent)
2	1.02 (0.96, 1.09)	1.04 (0.96, 1.12)	0.97 (0.87, 1.09)
3	1.06 (1.00, 1.13)	1.10 (1.02, 1.19)	0.99 (0.88, 1.11)
4	1.08 (1.01, 1.15)	1.10 (1.02, 1.19)	1.04 (0.93, 1.17)
5	1.21 (1.14, 1.29)	1.24 (1.14, 1.34)	1.16 (1.04, 1.30)

Abbreviations: BMI, body mass index; CI, confidence interval; CRC, colorectal cancer; DIS, dietary inflammation score; HR, hazard ratio; LIS, lifestyle inflammation score; NIH-AARP, National Institutes of Health-American Association for Retired Persons Diet and Health Study

* Inflammation score constructed as described in the text and Table 1; a higher score reflects a higher balance of pro-inflammatory exposures

† Covariates in the DIS Cox proportional hazards models were: age at entry (continuous), sex, race (Black, White, or other), education (less than high school and high school graduate, some college, or college graduate or higher), marital status (married or non-married), heart disease or history of stroke at baseline (yes/no), diabetes mellitus at baseline (yes/no), emphysema at baseline (yes/no), gallstone or gallbladder disease at baseline (yes/no), current hormone replacement therapy use (among women), family history of colorectal cancer in a first degree relative, history of colon polyp, smoking (current, former, or never), body mass index (in kg/m^2; continuous), alcohol intake (non-drinker, moderate-drinker, or heavy-drinker), physical activity level (exercises not at all or rarely, 1 – 2, or ≥ 3 times/wk), total energy intake (kcal/day), supplemental micronutrient score (calculated as described in Table 1); history of CRC in a first degree relative, self-reported heart disease diagnosis, age at entry, sex, and BMI were included in the SAS *STRATA* statement

Supplementary Table 5. Associations of the reverse HEI-2015* and the EDIP* with incident colorectal cancer overall, and by sex; the NIH-AARP Diet and Health Study (NIH-AARP; n = 453,465), 1995 – 2011

	Overall		Men		Women	
	HEI-2015†	EDIP‡	HEI-2015†	EDIP‡	HEI-2015†	EDIP‡
Quintiles	Adjusted HR (95% CI)	Adjusted HR (95% CI)	Adjusted HR (95% CI)	Adjusted HR (95% CI)	Adjusted HR (95% CI)	Adjusted HR (95% CI)
1	1.00 (Referent)	1.00 (Referent)	1.00 (Referent)	1.00 (Referent)	1.00 (Referent)	1.00 (Referent)
2	1.07 (1.00,1.14)	1.06 (0.99,1.13)	1.06 (0.98,1.15)	1.09 (1.01,1.18)	1.07 (0.95,1.20)	0.99 (0.88,1.11)
3	1.15 (1.08,1.23)	1.07 (1.01,1.14)	1.17 (1.08,1.26)	1.08 (1.00,1.17)	1.11 (0.99,1.24)	1.06 (0.95,1.18)
4	1.21 (1.13,1.29)	1.09 (1.02,1.16)	1.24 (1.15,1.34)	1.07 (0.99,1.15)	1.14 (1.02,1.28)	1.13 (1.01,1.26)
5	1.36 (1.27,1.45)	1.07 (1.00,1.14)	1.37 (1.26,1.48)	1.08 (1.00,1.16)	1.35 (1.21,1.51)	1.05 (0.94,1.17)

Abbreviations: CI, confidence interval; DIS, dietary inflammation score; EDIP, empirical dietary inflammation index; HEI, Healthy Eating Index 2015; HR, hazards ratio; LIS, lifestyle inflammation score; NIH-AARP, National Institutes of Health-American Association for Retired Persons Diet and Health Study

* The HEI was constructed as described by Krebs-Smith et al.(3), but the scoring was reversed such that a lower score was considered potentially higher risk; the EDIP was constructed as described by Tabung et al.(60) based on servings of intake

† Covariates in the HEI Cox proportional hazards models were: age at entry (continuous), sex, race (Black, White, or other), education (less than high school and high school graduate, some college, or college graduate or higher), marital status (married or non-married), heart disease or history of stroke at baseline (yes/no), diabetes mellitus at baseline (yes/no), emphysema at baseline (yes/no), gallstone or gallbladder disease at baseline (yes/no), current hormone replacement therapy use (among women), family history of colorectal cancer in a first degree relative, history of colon polyp, smoking (current, former, or never), body mass index (in kg/m^2; continuous), alcohol intake (non-drinker, moderate-drinker, or heavy-drinker), physical activity level (exercises not at all or rarely, 1 – 2, or ≥ 3 times/wk), and total energy intake (kcal/day); history of CRC in a first degree relative, self-reported heart disease diagnosis, age at entry, sex, and BMI were included in the SAS *STRATA* statement

‡ Covariates in the EDIP Cox proportional hazards models included those described in footnote 'b', except for alcohol intake

Supplementary Table 6. Associations of the individual components of the lifestyle inflammation score (LIS) with incident colorectal cancer overall, and by sex; the NIH-AARP Diet and Health Study (NIH-AARP; n = 453,465), 1995 – 2011

	Overall	Men	Women
Lifestyle factor*	Adjusted HR (95% CI)	Adjusted HR (95% CI)	Adjusted HR (95% CI)
Body mass index†			
Overweight vs. normal	1.11 (1.06, 1.16)	1.14 (1.07, 1.20)	1.07 (0.99, 1.16)
Obese vs. normal	1.24 (1.18, 1.31)	1.30 (1.21, 1.39)	1.15 (1.05, 1.26)
Physical activity level			
Exercises 1 – 2 times/wk vs. rarely/never exercises	0.92 (0.87, 0.98)	0.92 (0.85, 0.98)	0.93 (0.85, 1.01)
Exercises ≥ 3 times/wk vs. rarely/never exercises	0.85 (0.81, 0.90)	0.83 (0.78, 0.89)	0.88 (0.80, 0.96)
Alcohol use‡			
Moderate drinker vs. non-drinker	1.02 (0.98, 1.07)	1.02 (0.96, 1.08)	1.04 (0.96, 1.12)
Heavy drinker vs. non-drinker	1.23 (1.14, 1.33)	1.29 (1.17, 1.42)	1.13 (1.00, 1.28)
Smoking status			
Current smoker vs. never smoker	1.29 (1.21, 1.38)	1.20 (1.13, 1.27)	1.35 (1.22, 1.49)
Former smoker vs. never smoker	1.20 (1.15, 1.26)	1.25 (1.14, 1.36)	1.19 (1.10, 1.28)

Abbreviations: CI, confidence interval; DIS, dietary inflammation score; HR, hazards ratio; LIS, lifestyle inflammation score; NIH-AARP, National Institutes of Health-American Association for Retired Persons Diet and Health Study

* All lifestyle components were included in the Cox proportional hazards models and additionally included: age at entry (continuous), sex, race (Black, White, or other), education (less than high school and high school graduate, some college, or college graduate or higher), marital status (married or non-married), heart disease or history of stroke at baseline (yes/no), diabetes mellitus at baseline (yes/no), emphysema at baseline (yes/no), gallstone or gallbladder disease at baseline (yes/no), current hormone replacement therapy use (among women), family history of colorectal cancer in a first degree relative, history of colon polyp, total energy intake (kcal/day), former smoker (yes/no), and the equally-weighted DIS; history of CRC in a first degree relative, self-reported heart disease diagnosis, age at entry, and sex were included in the SAS *STRATA* statement

† Normal BMI: 18.5 – 24.99 kg/m2; Overweight BMI: 25 – 29.99 kg/m2; Obese BMI: ≥ 30 kg/m2

‡ Moderate drinker: 1 – 7 drinks/wk for women, 1 – 14 drinks/wk for men; heavy drinker: > 7 drinks/wk for women, > 14 drinks/wk drinks for men

Supplementary Table 7. Sensitivity analyses for the associations of the DIS and LIS with incident colorectal cancer in the NIH-AARP Diet and Health Study (NIH-AARP; n = 453,465), 1995 – 2011, excluding those who died or were diagnosed with CRC within two years from baseline

Score form	Inflammation score*	
	DIS†	LIS‡
	Adjusted HR (95% CI)	Adjusted HR (95% CI)
Continuous	1.04 (1.03, 1.05)	1.16 (1.12, 1.19)
Quintiles		
1	1.00 (Referent)	1.00 (Referent)
2	1.02 (0.95, 1.09)	1.12 (1.05, 1.20)
3	1.07 (1.00, 1.14)	1.18 (1.11, 1.27)
4	1.12 (1.05, 1.20)	1.21 (1.13, 1.29)
5	1.27 (1.18, 1.35)	1.37 (1.28, 1.47)

Abbreviations: CI, confidence interval; DIS, dietary inflammation score; HR, hazards ratio; LIS, lifestyle inflammation score; NIH-AARP, National Institutes of Health-American Association for Retired Person Diet and Health Study

* Inflammation scores constructed as described in the text and Table 1; a higher score reflects a higher balance of pro-inflammatory exposures

† Covariates in the DIS Cox proportional hazards models were: age at entry (continuous), sex, race (Black, White, or other), education (less than high school and high school graduate, some college, or college graduate or higher), marital status (married or non-married), heart disease or history of stroke at baseline (yes/no), diabetes mellitus at baseline (yes/no), emphysema at baseline (yes/no), gallstone or gallbladder disease at baseline (yes/no), current hormone replacement therapy use (among women), family history of colorectal cancer in a first degree relative, history of colon polyp, smoking (current, former, or never), body mass index (in kg/m^2; continuous), alcohol intake (non-drinker, moderate-drinker, or heavy-drinker), physical activity level (exercises not at all or rarely, 1 – 2, or ≥ 3 times/wk), and total energy intake (kcal/day); history of CRC in a first degree relative, self-reported heart disease diagnosis, age at entry, sex, and BMI were included in the SAS *STRATA* statement

‡ Covariates in the LIS Cox proportional Hazards models were: age at entry (continuous), sex, race (Black, White, or other), education (less than high school and high school graduate, some college, or college graduate or higher), marital status (married or non-married), heart disease or history of stroke at baseline (yes/no), diabetes mellitus at baseline (yes/no), emphysema at baseline (yes/no), gallstone or gallbladder disease at baseline (yes/no), current hormone replacement therapy use (among women), family history of colorectal cancer in a first degree relative, history of colon polyp, total energy intake (kcal/day), former smoker (yes/no), and the equally-weighted DIS; history of CRC in a first degree relative, self-reported heart disease diagnosis, age at entry, and sex were included in the SAS *STRATA* statement

Supplementary Table 8. Comparison of components and derivations of previously developed dietary and lifestyle inflammation scores (DII, EDIP, DIS, and LIS)

Description	Inflammation score			
	DII (61)	EDIP (4)	DIS*	LIS*
Anti-inflammatory components	Alcohol, β-carotene, caffeine, dietary fiber, folic acid, magnesium, thiamin, riboflavin, niacin, zinc, monounsaturated fats, polyunsaturated fats, Ω-3 fatty acids, Ω-6 fatty acids, selenium, isoflavones, flavan-3-ol, flavones, flavanols, flavanones, anthocyanins, green or black tea, garlic, onion, turmeric, thyme & oregano, pepper, rosemary, eugenol, ginger, saffron, and vitamins A, B-6, C, D, & E	Beer, wine, tea, coffee, dark-yellow vegetables, leafy green vegetables, snacks, fruit juice, and pizza	Leafy greens, tomatoes, apples and berries, deep yellow or orange vegetables and fruit, other fruits and real fruit juices, other vegetables, legumes, fish, poultry, high- and low-fat dairy, coffee and tea, nuts, supplement score	Moderate alcohol intake, moderate or heavy physical activity
Pro-inflammatory components	Vitamin B-12, iron, trans fat, carbohydrates, cholesterol, total energy intake, protein, saturated fat, and total fat	Processed meats, red meat, organ meat, fish (other than dark-meat fish), other vegetables (e.g., celery, mushrooms, green peppers, etc.), refined grains, high-energy beverages, low-energy beverages, and tomatoes	Red and organ meats, processed meats, added sugars, other fats, refined grains and starchy vegetables	Heavy alcohol intake, current tobacco use, overweight BMI, obese BMI
Derivation approach	Performed literature review of observational associations/intervention effects of 45 dietary components (mainly nutrients) with inflammation biomarkers	Used reduced rank regression to identify linear function of food groups that explain the most variation in inflammation biomarkers measured in subset of the Nurses' Health Study	Dietary components selected and grouped based on hypothesized contributions to inflammation; used multivariable linear regression to calculate β coefficients representing the average change in summary inflammation biomarker score per one standard deviation increase in a dietary component	Lifestyle components selected based on previous literature and biological plausibility (3–8,63); used multivariable linear regression to calculate β coefficients representing the average change in summary inflammation biomarker score with the presence of a lifestyle component
Inflammation biomarkers	IL-1β, IL-4, IL-6, IL-10, TNF-α, CRP	IL-6, TNF-α R2, CRP	IL-6, IL-8, IL-10, CRP	IL-6, IL-8, IL-10, CRP

Abbreviations: BMI, body mass index; CRP, C-reactive protein; DII, Dietary Inflammatory Index; DIS, Dietary Inflammation Score; EDIP, Empirical Dietary Inflammatory Pattern; IL, interleukin; LIS, lifestyle inflammation score; TNF-α R2, tumor necrosis factor-alpha receptor 2

* DIS and LIS components are defined in Table 1

References for Supplementary Tables

1. Furukawa S, Fujita T, Shimabukuro M, et al. Increased oxidative stress in obesity and its impact on metabolic syndrome. *J Clin Invest*. 2004;114(12):1752-1761. doi:10.1172/JCI21625
2. Calder PC, Ahluwalia N, Brouns F, et al. Dietary factors and low-grade inflammation in relation to overweight and obesity. *Br J Nutr*. 2011;106(S3):S5-S78. doi:10.1017/S0007114511005460
3. Wu D, Zhai Q, Shi X. Alcohol-induced oxidative stress and cell responses. *J Gastroenterol Hepatol*. 2006;21:26-29. doi:10.1111/j.1440-1746.2006.04589.x
4. Kumar Das S, Vasudevan DM. Alcohol-induced oxidative stress. *Life Sci*. 2007;81(3):177-187. https://ac-els-cdn-com.proxy.library.emory.edu/S002432050700358X/1-s2.0-S002432050700358X-main.pdf?_tid=97c2d41b-7a69-4f02-9415-df38ed8511f5&acdnat=1534710321_4f7c42d0b9718382d5000f33a769a18e. Accessed August 19, 2018.
5. Mathews MJ, Liebenberg L, Mathews EH. The mechanism by which moderate alcohol consumption influences coronary heart disease. *Nutr J*. 2015;14(1):33. doi:10.1186/s12937-015-0011-6
6. McCarty M. Interleukin-6 as a central mediator of cardiovascular risk associated with chronic inflammation, smoking, diabetes, and visceral obsesity: down-regulation with essential fatty acids, ethanol and pentoxifylline. *Med Hypotheses*. 1999;52(5):465-477. https://ac-els-cdn-com.proxy.library.emory.edu/S0306987797906848/1-s2.0-S0306987797906848-main.pdf?_tid=8caa11c0-7491-4684-8d9d-3b986892707e&acdnat=1537892830_3167c925a76d95eacafa5e7e535dccc0. Accessed September 25, 2018.
7. Gomez-Cabrera M-C, Domenech E, Viña J. Moderate exercise is an antioxidant: upregulation of antioxidant genes by training. *Free Radic Biol Med*. 2008;44(2):126-131. doi:10.1016/j.freeradbiomed.2007.02.001
8. van der Vaart H, Postma D, Timens W, Ten Hacken N. Acute effects of cigarette smoke on inflammation and oxidative stress: a review. *Thorax*. 2004;59:713-721. doi:10.1136/thx.2003.012468

9. Hussain T, Tan B, Liu G, et al. Modulatory mechanism of polyphenols and Nrf2 Signaling Pathway in LPS challenged pregnancy disorders. *Oxid Med Cell Longev*. 2017;2017:1-14. doi:10.1155/2017/8254289

10. Johnson M, Pace RD, Mcelhenney WH. Green leafy vegetables in diets with a 25:1 omega-6/omega-3 fatty acid ratio modify the erythrocyte fatty acid profile of spontaneously hypertensive rats. *Lipids Health Dis*. 2018;17(140). doi:10.1186/s12944-018-0723-7

11. Sommerburg O, E Keunen JE, Bird AC, G M van Kuijk FJ. Fruits and vegetables that are sources for lutein and zeaxanthin: the macular pigment in human eyes. *Br J Ophthalmol*. 1998;82:907-910. https://www.ncbi.nlm.nih.gov/pmc/articles/PMC1722697/pdf/v082p00907.pdf. Accessed February 1, 2017.

12. Brown A, Hu Frank. Dietary modulation of endothelial function: implications for cardiovascular disease. *Am J Clin Nutr*. 2001;73:673-686. https://academic.oup.com/ajcn/article-abstract/73/4/673/4737337. Accessed August 9, 2018.

13. Guardia T, Rotelli AE, Osvaldo Juarez A, Pelzer LE. Anti-inflammatory properties of plant flavonoids. Effects of rutin, quercetin and hesperidin on adjuvant arthritis in rat. *Farm*. 2001;56(2001):683-687. www.elsevier.com/locate/farmac. Accessed February 1, 2017.

14. Obeid R, Kirsch SH, Kasoha M, Eckert R, Herrmann W. Concentrations of unmetabolized folic acid and primary folate forms in plasma after folic acid treatment in older adults. *Metabolism*. 2011;60(5):673-680. doi:10.1016/j.metabol.2010.06.020

15. Kelly K, Kennelly J, Ordonez M, et al. Excess folic acid increases lipid storage, weight gain, and adipose tissue inflammation in high fat diet-fed rats. *Nutrients*. 2016;8(10):594. doi:10.3390/nu8100594

16. Du S-Y, Zhang Y-L, Bai R-X, Ai Z-L, Xie B-S, Yang H-Y. Lutein prevents alcohol-induced liver disease in rats by modulating oxidative stress and inflammation. *Int J Clin Exp Med*. 2015;8(6):8785-8793. www.ijcem.com. Accessed February 1, 2017.

17. Wang M-X, Jiao J-H, Li Z-Y, Liu R-R, Shi Q, Ma L. Lutein supplementation reduces plasma lipid peroxidation and C-reactive protein in healthy nonsmokers. *Atherosclerosis*. 2013;227(2):380-385. doi:10.1016/j.atherosclerosis.2013.01.021
18. Johnson EJ. Role of lutein and zeaxanthin in visual and cognitive function throughout the lifespan. *Nutr Rev*. 2014;72(9):605-612. doi:10.1111/nure.12133
19. Nidhi B, Sharavana G, Ramaprasad TR, Vallikannan B. Lutein derived fragments exhibit higher antioxidant and anti-inflammatory properties than lutein in lipopolysaccharide induced inflammation in rats. *Food Funct*. 2015;6(2):450-460. doi:10.1039/c4fo00606b
20. Rao A V. Lycopene, tomatoes, and the prevention of coronary heart disease. *Exp Biol Med*. 2002;227(10):908-913. http://www.ncbi.nlm.nih.gov/pubmed/12424333. Accessed February 1, 2017.
21. Burton-Freeman BM, Sesso HD. Whole food versus supplement: comparing the clinical evidence of tomato intake and lycopene supplementation on cardiovascular risk factors. *Adv Nutr*. 2014;5(5):457-485. doi:10.3945/an.114.005231
22. Jacob K, Periago MJ, Böhm V, Berruezo GR. Influence of lycopene and vitamin C from tomato juice on biomarkers of oxidative stress and inflammation. *Br J Nutr*. 2017;99(01):137-146. doi:10.1017/S0007114507791894
23. Markovits N, Amotz A, Levy Y. The effect of tomato-derived lycopene on low carotenoids and enhanced systemic inflammation and Oxidation in severe Obesity. *IMAJ*. 2009;11:598-601.
24. Prior RL, Gu L, Wu X, et al. Plasma antioxidant capacity changes following a meal as a measure of the ability of a food to alter in vivo antioxidant status. *J Am Coll Nutr*. 2007;26(2):170-181. doi:10.1080/07315724.2007.10719599
25. Espley R V, Butts CA, Laing WA, et al. Dietary flavonoids from modified apple reduce inflammation markers and modulate gut microbiota in mice. *J Nutr*. 2014;144(2):146-154. doi:10.3945/jn.113.182659

26. Codoñer-Franch P, Betoret E, Betoret N, López-Jaén AB, Valls-Belles V, Fito P. Dried apples enriched with mandarin juice by vacuum impregnation improve antioxidant capacity and decrease inflammation in obese children. *Nutr Hosp*. 2013;28(3):1177-1183. doi:10.3305/nh.2013.28.4.6580
27. Kaulmann A, Bohn T. Carotenoids, inflammation, and oxidative stress—implications of cellular signaling pathways and relation to chronic disease prevention. *Nutr Res*. 2014;34(11):907-929. doi:10.1016/j.nutres.2014.07.010
28. Ghanim H, Mohanty P, Pathak R, Chaudhuri A, Chang LS, Dandona P. Orange juice or fructose intake does not induce oxidative and inflammatory response. *Diabetes Care*. 2007;30(6):1406-1411. doi:10.2337/dc06-1458
29. Knekt P, Kumpulainen J, Järvinen R, et al. Flavonoid intake and risk of chronic diseases. *Am J Clin Nutr*. 2002;76(3):560-568.
30. Fortis-Barrera Á, Alarcón-Aguilar FJ, Banderas-Dorantes T, et al. Cucurbita ficifolia Bouché (Cucurbitaceae) and D-chiro-inositol modulate the redox state and inflammation in 3T3-L1 adipocytes. *J Pharm Pharmacol*. 2013;65(10):1563-1576. doi:10.1111/jphp.12119
31. Sharma D, Rawat I, Goel HC. Anticancer and anti-inflammatory activities of some dietary cucurbits. *Indian J Exp Biol*. 2015;53(4):216-221. http://www.ncbi.nlm.nih.gov/pubmed/26011982.
32. Hale LP, Chichlowski M, Trinh CT, Greer PK. Dietary supplementation with fresh pineapple juice decreases inflammation and colonic neoplasia in IL-10-deficient mice with colitis. *Inflamm Bowel Dis*. 2010;16(12):2012-2021. doi:10.1002/ibd.21320
33. Böhm F, Settergren M, Pernow J. Vitamin C blocks vascular dysfunction and release of interleukin-6 induced by endothelin-1 in humans in vivo. *Atherosclerosis*. 2007;190(2):408-415. doi:10.1016/j.atherosclerosis.2006.02.018
34. Alam MA, Subhan N, Rahman MM, Uddin SJ, Reza HM, Sarker SD. Effect of citrus flavonoids, naringin and naringenin, on metabolic syndrome and their mechanisms of action. *Adv Nutr*. 2014;5(4):404-417. doi:10.3945/an.113.005603

35. Jia Q, Cheng W, Yue Y, et al. Cucurbitacin E inhibits TNF-α-induced inflammatory cytokine production in human synoviocyte MH7A cells via suppression of PI3K/Akt/NF-κB pathways. *Int Immunopharmacol*. 2015;29(2):884-890. doi:10.1016/j.intimp.2015.08.026
36. Zitvogel L, Pietrocola F, Kroemer G. Nutrition, inflammation and cancer. *Nat Immunol*. 2017;18(8):843-850. doi:10.1038/ni.3754
37. Hartman TJ, Albert PS, Zhang Z, et al. Consumption of a legume-enriched, low-glycemic index diet is associated with biomarkers of insulin resistance and inflammation among men at risk for colorectal cancer. *J Nutr*. 2010;140(1):60-67. doi:10.3945/jn.109.114249
38. Simopoulos AP. Omega-3 fatty acids in inflammation and autoimmune diseases. *J Am Coll Nutr*. 2002;21(6):495-505. http://www.ncbi.nlm.nih.gov/pubmed/12480795.
39. Calder PC. Omega-3 fatty Acids and inflammatory processes. *Nutrients*. 2010;2(3):355-374. doi:10.3390/nu2030355
40. Giugliano D, Ceriello A, Esposito K. The effects of diet on inflammation: emphasis on the metabolic syndrome. *J Am Coll Cardiol*. 2006;48(4):677-685. doi:10.1016/j.jacc.2006.03.052
41. van Woudenbergh GJ, Kuijsten A, Tigcheler B, et al. Meat consumption and its association with C-reactive protein and incident type 2 diabetes: the Rotterdam Study. *Diabetes Care*. 2012;35(7):1499-1505. doi:10.2337/dc11-1899
42. van Bussel BC, Henry RM, Ferreira I, et al. A Healthy diet is associated with less endothelial dysfunction and less low-grade inflammation over a 7-Year period in adults at risk of cardiovascular disease. *J Nutr*. 2015;145(3):532-540. doi:10.3945/jn.114.201236
43. Song M, Garrett WS, Chan AT. Nutrients, foods, and colorectal cancer prevention. *Gastroenterology*. 2015;148(6):1244-1260.e16. doi:10.1053/j.gastro.2014.12.035
44. Minihane AM, Vinoy S, Russell WR, et al. Low-grade inflammation, diet composition and health: current research evidence and its translation. *Br J Nutr*. 2015;114(7):999-1012. doi:10.1017/S0007114515002093

45. Lopez-Garcia E, Schulze MB, Meigs JB, et al. Consumption of trans fatty acids is related to plasma biomarkers of inflammation and endothelial dysfunction. *J Nutr*. 2005;135(3):562-566. https://academic.oup.com/jn/article-abstract/135/3/562/4663700. Accessed August 19, 2018.
46. Ley SH, Sun Q, Willett WC, et al. Associations between red meat intake and biomarkers of inflammation and glucose metabolism in women. *Am J Clin Nutr*. 2014;99(2):352-360. doi:10.3945/ajcn.113.075663
47. White DL, Collinson A. Red meat, dietary heme iron, and risk of type 2 diabetes: the involvement of advanced lipoxidation endproducts. *Adv Nutr*. 2013;4(4):403-411. doi:10.3945/an.113.003681
48. Kallio P, Kolehmainen M, Laaksonen DE, et al. Dietary carbohydrate modification induces alterations in gene expression in abdominal subcutaneous adipose tissue in persons with the metabolic syndrome: the FUNGENUT Study. *Am J Clin Nutr*. 2007;85(5):1417-1427. https://academic.oup.com/ajcn/article-abstract/85/5/1417/4754402. Accessed August 10, 2018.
49. Guzik T, Korbut R, Adamek-Guzik T. Nitric oxide and superoxide in inflammation and immune regulation. *J Physiol Pharmacol*. 2003;54(4):469-487. www.jpp.krakow.pl. Accessed September 25, 2018.
50. Ludwig DS. The glycemic index: physiological mechanisms relating to obesity, diabetes, and cardiovascular disease. *JAMA*. 2002;287(18):2414-2423. doi:10.1001/jama.287.18.2414
51. Govers M, Termont D, Lapre J. Calcium in milk products precipitates intestinal fatty acids and secondary bile acids and thus inhibits colonic cytotoxicity in humans. *Cancer Res*. 1996;56(14):3270-3275. http://cancerres.aacrjournals.org/content/56/14/3270.full-text.pdf. Accessed August 18, 2018.
52. Dash C, Goodman M, Flanders WD, Mink PJ, McCullough ML, Bostick RM. Using pathway-specific comprehensive exposure scores in epidemiology: application to oxidative balance in a pooled case-control study of incident, sporadic colorectal adenomas. *Am J Epidemiol*. 2013;178(4):610-624. doi:10.1093/aje/kwt007
53. Kratz M, Baars T, Guyenet S. The relationship between high-fat dairy consumption and obesity, cardiovascular, and metabolic disease. *Eur J Nutr*. 2013;52(1):1-24. doi:10.1007/s00394-012-0418-1

54. Dower JI, Geleijnse JM, Gijsbers L, Schalkwijk C, Kromhout D, Hollman PC. Supplementation of the pure flavonoids epicatechin and quercetin affects some biomarkers of endothelial dysfunction and inflammation in (pre)hypertensive adults: a randomized double-blind, placebo-controlled, crossover trial. *J Nutr*. 2015;145(7):1459-1463. doi:10.3945/jn.115.211888

55. Park JB. Javamide-II found in coffee is better than caffeine at suppressing TNF-α production in PMA/PHA-treated lymphocytic jurkat cells. *J Agric Food Chem*. 2018;66(26):6782-6789. doi:10.1021/acs.jafc.8b01885

56. Wall R, Ross RP, Fitzgerald GF, Stanton C. Fatty acids from fish: the anti-inflammatory potential of long-chain omega-3 fatty acids. *Nutr Rev*. 2010;68(5):280-289. doi:10.1111/j.1753-4887.2010.00287.x

57. Casas-Agustench P, Bulló M, Salas-Salvadó J. Nuts, inflammation and insulin resistance. *Asia Pac J Clin Nutr*. 2010;19(1):124-130.

58. Chassaing B, Koren O, Goodrich JK, et al. Dietary emulsifiers impact the mouse gut microbiota promoting colitis and metabolic syndrome. *Nature*. 2015;519(7541):92-96. doi:10.1038/nature14232

59. Krebs-Smith SM, Pannucci TE, Subar AF, et al. Update of the Healthy Eating Index: HEI-2015. *J Acad Nutr Diet*. 2018;118(9):1591-1602. doi:10.1016/j.jand.2018.05.021

60. Tabung FK, Smith-Warner SA, Chavarro JE, et al. Development and validation of an empirical dietary inflammatory index. *J Nutr*. 2016;146(8):1560-1570. doi:10.3945/jn.115.228718

61. Shivappa N, Steck SE, Hurley TG, Hussey JR, Hébert JR. Designing and developing a literature-derived, population-based dietary inflammatory index. *Public Health Nutr*. 2014;17(8):1689-1696. doi:10.1017/S1368980013002115

62. Tabung FK, Smith-Warner SA, Chavarro JE, et al. Development and Validation of an Empirical Dietary Inflammatory Index. *J Nutr*. 2016;146(8):1560-1570. doi:10.3945/jn.115.228718

63. Calder PC, Ahluwalia N, Brouns F, et al. Dietary factors and low-grade inflammation in relation to overweight and obesity. *Br J Nutr*. 2011;106(S3):S5-S78. doi:10.1017/S0007114511005460
